# New biosensor for the early detection of tPSA in the serum of prostate cancer patients using a Tb-anthracene-9-carboxaldehyde complex embedded in a modified cellulose polymer thin film

**DOI:** 10.1039/d6ra01637e

**Published:** 2026-06-03

**Authors:** Marwa T. Abo Gabal, Mohamed A. El-Desouky, Demiana H. Hanna, Mohamed S. Attia

**Affiliations:** a Chemistry Department, Faculty of Science, Cairo University Giza 12613 Egypt mgaber@imamu.edu.sa; b Chemistry Department, College of Science, Imam Mohammad Ibn Saud Islamic University (IMSIU) Riyadh 11623 Saudi Arabia

## Abstract

This study reports the development and validation of a novel optical biosensor for the early detection of total prostate-specific antigen (tPSA) in serum samples from prostate cancer patients, utilizing a terbium-anthracene-9-carboxaldehyde (Tb-A9C) complex embedded within an epoxy-functionalized carboxymethyl cellulose (CMC) polymer thin film. The sensor was systematically optimized by investigating solvent effects and metal-to-ligand molar ratios, with dimethyl sulfoxide (DMSO) and a 1 : 1 (Tb : A9C) stoichiometry identified as optimal conditions yielding maximum luminescence intensity at 545 nm. Surface characterization *via* SEM confirmed successful layer-by-layer assembly, showing transformation from a cracked polymer surface to a smooth sensor-embedded film, followed by dendritic antibody immobilization patterns, while FTIR spectroscopy validated each fabrication stage through characteristic functional group signatures. Upon functionalization with anti-PSA monoclonal antibodies *via* glutaraldehyde crosslinking, the thin film sensor exhibited concentration-dependent luminescence quenching upon tPSA binding, enabling quantitative detection across a linear range of 0.025–0.30 ng mL^−1^ with an exceptionally low detection limit of 0.0159 ng mL^−1^ and excellent coefficient of determination (*R*^2^ = 0.995). The biosensor demonstrated outstanding accuracy and precision in both intra-day and inter-day assessments using clinical serum samples from prostate cancer patients, showing strong agreement with standard ELISA methods (sensitivity: 100%, specificity: 100%). Molecular docking and 100-ns molecular dynamics simulations revealed stable binding of the Tb-A9C complex within the PSA pocket C3 (Δ*G* = −7.5 kcal mol^−1^), with persistent hydrogen bonding networks and minimal structural fluctuations confirming specific and stable complex formation. The superior photophysical properties of the terbium complex, including large Stokes shift, long luminescence lifetime enabling time-gated detection, combined with the stabilizing CMC polymer matrix and computational validation of binding stability, establish this Tb-A9C thin film biosensor as a simple, sensitive, stable, and cost-effective alternative to conventional diagnostic methods for early prostate cancer detection and clinical monitoring.

## Abstract 2

Optical Tb-A9C/CMC biosensor for early tPSA detection in serum from prostate cancer patients: development & validation.

## Graphical abstract

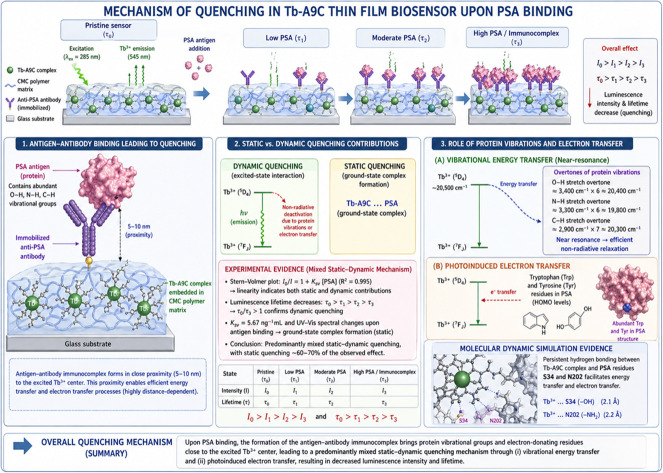

## 1 Introduction

Cancer remains a major cause of premature death globally.^1^ In 2020, GLOBOCAN estimated approximately 19.3 million new cancer cases and 10.3 million deaths worldwide.^1^ Among men, prostate cancer was the second most diagnosed cancer (1.4 million new cases) and the sixth leading cause of cancer mortality, responsible for about 375 000 deaths.^2^

In Egypt, the national incidence rate of prostate cancer averages 4.27 per 100 000 men, with 4767 new cases and 2227 deaths recorded in 2020.^3,4^ The risk increases significantly after age 50, with the highest occurrence in the 60–70 age bracket.^5,6^

The diagnostic landscape for prostate cancer (PCa) presents a critical paradox: while localized cases have a nearly 100% five-year survival rate, this drops to 30% following metastasis, underscoring the urgent need for early and accurate detection.^6^ The Prostate-Specific Antigen (PSA) test, introduced in the 1990s, remains widely used but is highly debated due to false positives and associated complications from subsequent biopsies.^7^ Other imaging techniques, such as Transrectal Ultrasonography (TRUS) and Multiparametric Magnetic Resonance Imaging (mpMRI), suffer from limited sensitivity, lack of real-time capability, or require specialized training.^8,9^ The current global standard for definitive diagnosis—transrectal or transperineal prostate biopsy—is invasive and carries risks of infection and bleeding. Moreover, TRUS-guided biopsies misdiagnose over half of men with clinically significant PCa, highlighting an urgent need for better non-invasive methods.^10^

These limitations have driven research toward biomarkers—measurable indicators of biological states.^11,12^ Protein biomarkers, particularly dysregulated receptor proteins, are especially valuable for precise diagnosis and monitoring.^13^ While conventional techniques like ELISA and mass spectrometry exist, they are often hampered by complex equipment, lengthy sample preparation, and an inability to detect very low biomarker concentrations without disrupting the protein's native state.^14^ Consequently, there is a major focus on developing novel luminescent-based techniques for rapid, sensitive, and field-ready PSA detection.^14^

A promising avenue lies in optical biosensors,^15^ particularly those based on lanthanide ions such as terbium (Tb^3+^).^16^ Tb^3+^ complexes possess distinct advantages: long-lived luminescence (millisecond scale), sharply defined emission peaks, and large Stokes shifts. These properties enable time-gated detection, which filters out short-lived autofluorescence from biological samples, greatly improving detection clarity.^17^ The strategic selection of Anthracene-9-Carboxaldehyde (A9C) as a ligand is pivotal due to its superior light-harvesting ability as a polycyclic aromatic hydrocarbon.^18^ Upon complexation with Tb^3+^, A9C functions as an “antenna”, absorbing light and efficiently transferring energy to the Tb^3+^ ion, which then emits a sensitized and significantly enhanced luminescent signal.^19,20^

The proposed biosensor integrates the Tb-A9C complex with carboxymethyl cellulose (CMC)—a versatile, biodegradable polymer known for biocompatibility and excellent film-forming ability.^21^ The synergy between the luminescent probe and the CMC matrix is intended to create a highly sensitive and durable biosensing platform.^46^

The Tb-A9C complex differs from conventional lanthanide-based sensors in several critical aspects. First, unlike most reported lanthanide biosensors that employ macrocyclic chelators (*e.g.*, DOTA, DTPA) requiring multi-step synthesis, our system uses a simple, commercially available ligand that forms a stable complex with Tb^3+^ in a single coordination step. Second, the antenna efficiency of A9C provides superior sensitization of Tb^3+^ luminescence compared to traditional β-diketonate ligands. Third, the integration of this complex into an epoxy-functionalized CMC matrix represents an unprecedented combination that enables stable antibody immobilization while maintaining the photophysical integrity of the lanthanide probe.^18–21^

The Tb-A9C complex offers: (i) a large Stokes shift (>200 nm) minimizing spectral overlap; (ii) millisecond-scale luminescence lifetime enabling time-gated detection; (iii) excellent photostability; (iv) simple one-pot synthesis; and (v) tunable emission properties through solvent and stoichiometry optimization. The epoxy-functionalized CMC matrix provides: (i) a hydrophilic environment maintaining antibody native conformation; (ii) reactive epoxy groups for covalent glutaraldehyde-mediated antibody immobilization; (iii) mechanical stability and optical transparency; (iv) protection of the Tb-A9C complex from external quenchers; and (v) biodegradable and biocompatible properties suitable for clinical applications^21–26^

This research is therefore dedicated to the development and characterization of a novel optical biosensor utilizing the Tb-A9C complex. The primary novelty lies in the first-time application of this specific complex for PSA biomarker detection. The approach integrates the high specificity of antibody-based recognition with the superior sensitivity of lanthanide luminescence. A key objective is to validate the sensor's performance within complex prostate cancer patient samples, thereby directly addressing the critical challenge of matrix interference in real-world diagnostic scenarios.

## 2 Materials and methods

### 2.1 Instruments

A comprehensive array of analytical instruments was employed throughout this study to characterize the synthesized materials and evaluate the performance of the developed biosensor. UV-visible absorption spectra were recorded using a Jenway spectrophotometer Model 6850 UV/Visible double-beam instrument equipped with a xenon flash lamp, offering a spectral range of 190–1100 nm as the light source. All luminescence measurements, including emission spectra and lifetime decay profiles, were performed on a high-resolution spectrofluorometer (Edinburgh Instruments FS5) with a spectral range extending up to 1650 nm and the capability to measure fluorescence lifetimes down to 25 ps. The pH of all prepared solutions was measured and adjusted using a Jenway 3040 pH meter. For sample preparation involving separation or purification steps, a Daihan Scientific centrifuge (CF-10 model) was utilized. Surface morphological characterization of the fabricated thin films was conducted using high-resolution Scanning Electron Microscopy (SEM) on an FEI Quanta FEG 250 instrument. The identification of functional groups and verification of chemical modifications at each fabrication stage were performed using Attenuated Total Reflectance-Fourier Transform Infrared (ATR-FTIR) spectroscopy on a THERMO NICOLET 50 spectrometer. Thin film deposition was achieved using a spin coater, and sample heating and stirring were facilitated by a hot plate equipped with magnetic stirring capability.

### 2.2 Materials

All chemical reagents and biological materials utilized in this investigation were of analytical grade and employed without further purification. Terbium nitrate pentahydrate (Tb(NO_3_)_3_·5H_2_O) served as the source of trivalent terbium ions (Tb^3+^) for complex synthesis. The organic ligand, anthracene-9-carboxaldehyde (C_15_H_10_O), was selected as the antenna molecule for sensitizing terbium luminescence. A systematic solvent screening study was conducted using four high-purity solvents: anhydrous dimethyl sulfoxide (DMSO, ≥99.9%), dimethyl formamide (DMF, HPLC grade), acetonitrile (ACN, HPLC grade), and absolute ethanol (EtOH, ≥99.8%), all acquired from Sigma-Aldrich or Fisher Scientific as specified. The biological recognition elements comprised Prostate-Specific Antigen (PSA, 150 ng mL^−1^) and monoclonal antibody against PSA (0.5 mg mL^−1^), both procured from MyBioSource (MBS9510722). For the fabrication of the polymer support matrix, sodium carboxymethyl cellulose (CMC) with an average molecular weight range of 250 000–700 000 g mol^−1^ (CAS No. 9004-32-4, CP Kelco Cekol® 30000) was utilized. Epichlorohydrin (≥99% purity, CAS No. 106-89-8, Qualikems Fine Chemicals Pvt. Ltd, India) served as the crosslinking agent for polymer activation, while glutaraldehyde solution (25%, C_5_H_8_O_2_, CAS No. 111-30-8, Fisher Scientific, USA) was employed as a bifunctional crosslinker for antibody immobilization. Human serum samples were obtained from two medical institutions in Cairo, Egypt: Al-Kasr El-Aini New Teaching Hospital and Ain Shams Specialized Hospital. The sample collection process strictly adhered to protocols approved by the World Health Organization (WHO) for human specimen collection. All experimental procedures were conducted in compliance with the ethical guidelines established by the Egyptian Ministry of Health and Population and received approval from the Ethics Committee of the Faculty of Pharmacy, British University in Egypt. Written informed consent was secured from all participants prior to their inclusion in the study.

### 2.3 Methodology

#### 2.3.1 Solvent optimization for complex formation

To identify the optimal solvent medium for sensor fabrication, a comprehensive solvent effect study was conducted by systematically evaluating four different solvents: anhydrous dimethyl sulfoxide (DMSO), dimethyl formamide (DMF), acetonitrile (ACN), and absolute ethanol (EtOH). This investigation aimed to determine the most suitable solvent environment that would ensure maximum luminescence efficiency of the Tb-A9C complex. Terbium stock solutions were prepared by accurately dissolving 0.01589 grams of terbium nitrate pentahydrate in 10 mL of each solvent individually, yielding four distinct terbium solutions. Concurrently, ligand stock solutions were prepared by dissolving 0.021 grams of anthracene-9-carboxaldehyde in 10 mL of each of the four solvents separately. For each solvent system, the corresponding pair of stock solutions was combined in a 1 : 1 molar ratio (metal : ligand) in 10 mL of the respective solvent. The luminescence emission intensity of each resulting mixture was immediately measured using the spectrofluorometer. Comparative analysis of the emission spectra identified anhydrous dimethyl sulfoxide (DMSO) as the solvent producing the highest luminescence intensity, establishing it as the most effective medium for sensor fabrication. Consequently, DMSO was selected and employed for all subsequent sensor fabrication steps and analytical applications throughout this study.

#### 2.3.2 Optimization of metal-to-ligand molar ratio

The stoichiometric ratio between terbium ions and anthracene-9-carboxaldehyde ligand was systematically optimized to achieve maximum luminescence output. Three different molar ratios of metal to ligand, namely 1 : 1, 1 : 2, and 1 : 3 (Tb : anthracene-9-carboxaldehyde), were prepared in DMSO as the optimized solvent. Each solution was allowed to equilibrate, and the luminescence emission intensity was measured using the spectrofluorometer. Analysis of the emission spectra revealed that the 1 : 1 molar ratio produced the highest luminescence intensity across all characteristic terbium emission peaks, particularly at the dominant 545 nm transition. This ratio was therefore identified as the optimal stoichiometry for complex formation, providing the ideal coordination environment that maximizes energy transfer efficiency from the anthracene-9-carboxaldehyde ligand to the Tb^3+^ center while minimizing non-radiative decay pathways.

#### 2.3.3 Sensor fabrication

Based on the optimized parameters identified through solvent and molar ratio studies, the Tb-A9C complex was fabricated under controlled conditions. Working solutions were prepared at concentrations of 1.0 × 10^−4^ mol L^−1^ for terbium and 2.0 × 10^−4^ mol L^−1^ for anthracene-9-carboxaldehyde in DMSO. These solutions were combined in the optimized 1 : 1 molar ratio to form the luminescent complex, which served as the active sensing element for subsequent thin film incorporation and analytical applications.

#### 2.3.4 Preparation of working solutions for biological components

The Prostate-Specific Antigen (PSA) antigen working solutions were prepared by dissolving the contents of the commercial vial (150 ng mL^−1^) in 2 mL of deionized water. From this stock solution, serial dilutions were performed using deionized water to achieve various PSA concentrations required for calibration and analytical measurements. The anti-PSA monoclonal antibody working solution was prepared by diluting the commercial preparation (0.5 mg mL^−1^) in 1 mL of deionized water. All prepared biological solutions were stored at 4 °C when not in use to maintain their stability and biological activity throughout the experimental period.

#### 2.3.5 Preparation of epoxy-functionalized carboxymethyl cellulose polymer

The polymer support matrix was prepared through chemical modification of carboxymethyl cellulose (CMC) using epichlorohydrin as a crosslinking and activation agent. Three grams of carboxymethyl cellulose were dissolved (suspended) in 100 mL of deionized water with continuous stirring to ensure complete dispersion. To this suspension, 4 mL of epichlorohydrin were added, and the activation reaction was conducted at 65 °C for 3 hours in a thermostatically controlled water bath. Throughout the reaction period, the pH of the reaction mixture was maintained under basic conditions by the dropwise addition of dilute sodium hydroxide solution, which promotes the reaction of epichlorohydrin with the hydroxyl groups of the CMC polymer backbone, facilitating effective crosslinking and epoxy functionalization. Following completion of the activation reaction, the solution was allowed to cool to room temperature, then cast into a Petri dish and left to dry overnight at 25 °C, yielding the epoxy-functionalized CMC polymer film.

#### 2.3.6 Preparation of epoxy-functionalized CMC thin film embedded with terbium-anthracene-9-carboxaldehyde complex

The luminescent composite thin film was prepared by incorporating the pre-formed Tb-A9C complex into the epoxy-functionalized CMC matrix. Six milliliters of the prepared epoxy-functionalized CMC polymer solution were thoroughly mixed with 1 mL of the Tb-A9C complex solution for approximately 5 minutes to ensure homogeneous distribution of the luminescent complex within the polymer matrix. The resulting mixture was then deposited as a thin film using a spin coater operating at 500 rpm to achieve uniform thickness and surface coverage. Subsequently, the spin-coated film was cast in a Petri dish and allowed to dry overnight at 25 °C, producing a stable, free-standing thin film with the luminescent terbium complex uniformly embedded within the crosslinked cellulose network.

#### 2.3.7 Immobilization of anti-PSA antibodies onto the thin film surface

The surface of the prepared thin film was functionalized to enable covalent immobilization of anti-PSA monoclonal antibodies. A section of the thin film was affixed to a clean microscope slide using phosphate-buffered saline (PBS) as an adhesive and hydration medium. The surface was then treated by applying drops of glutaraldehyde solution (25%), which serves as a bifunctional crosslinking agent. One aldehyde group of glutaraldehyde reacts with the epoxy and hydroxyl functionalities on the modified CMC film surface, while the other aldehyde group remains available for subsequent reaction with amine groups on the antibody molecules. The glutaraldehyde solution was allowed to react with the film surface for 10 minutes at room temperature. Following this activation step, the primary anti-PSA monoclonal antibody solution was applied dropwise onto the glutaraldehyde-functionalized surface, enabling covalent attachment through Schiff base formation between the free aldehyde groups and the lysine residues of the antibody. The prepared antibody-immobilized thin film slides were stored at 4 °C for subsequent analytical applications.

#### 2.3.8 Calibration curve construction

The analytical performance of the fabricated thin film biosensor for PSA antigen detection was evaluated by constructing a calibration curve. Standard PSA solutions (50 µL) at varying concentrations were incubated with the antibody-immobilized thin film sensor to allow specific antigen–antibody binding. Following incubation, fluorescence measurements were performed using excitation and emission wavelengths of 320 nm and 545 nm, respectively, which correspond to the optimal excitation of the anthracene-9-carboxaldehyde antenna and the characteristic terbium emission maximum. To prevent sample carryover and cross-contamination between measurements, the quartz cuvette was thoroughly rinsed with deionized water after each measurement. A calibration plot was constructed by plotting the normalized fluorescence intensity, expressed as (*F*_0_/*F*) − 1, at 545 nm against the corresponding PSA concentration, where *F*_0_ represents the fluorescence intensity in the absence of PSA and *F* represents the intensity after antigen binding.

#### 2.3.9 Clinical sample collection and analysis

Ten venous blood samples were obtained from confirmed prostate cancer patients attending Al-Kasr El-Aini New Teaching Hospital and Ain Shams Specialized Hospital in Cairo, Egypt. All patients had histopathologically confirmed prostate cancer, following WHO-approved protocols for human specimen collection. All experimental procedures involving human samples were conducted in full compliance with the ethical guidelines of the Egyptian Ministry of Health and Population and were approved by the Ethics Committee of the Faculty of Pharmacy, British University in Egypt, with written informed consent obtained from all participants.

For the analysis of clinical samples, the intra-day and inter-day precision of the fluorometric assay were assessed using human serum samples. Serum samples containing varying concentrations of PSA were introduced to 1 mL of the sensor solution or directly incubated with the thin film sensor, and fluorescence intensity was measured at the emission wavelength of 545 nm. For intra-day repeatability assessment, triplicate measurements for each concentration level were acquired within a single analytical run, with thorough rinsing of the quartz cuvette between measurements to prevent carryover. For inter-day reproducibility assessment, the entire analytical procedure, including sensor–serum combinations, was performed in triplicate on three separate days under identical experimental conditions to evaluate the robustness and consistency of the method over time.

#### 2.3.10 Calculation of diagnostic performance metrics

The diagnostic efficacy of the proposed biosensor was evaluated by calculating key performance indicators including sensitivity, specificity, positive predictive value (PPV), and negative predictive value (NPV). These metrics were derived using the following standard formulas:

• Sensitivity = [*A*/(*A* + *B*)] × 100%, where *A* represents the number of true positive results and *B* represents the number of false negative results.

• Specificity = [*C*/(*C* + *D*)] × 100%, where *C* represents the number of true negative results and *D* represents the number of false positive results.

• Positive predictive value (PPV) = [*A*/(*A* + *D*)] × 100%.

• Negative predictive value (NPV) = [*C*/(*C* + *B*)] × 100%.

• Disease prevalence = (*T*_disease/total) × 100%, where *T*_disease is the number of individuals confirmed with prostate cancer and total is the total number of individuals tested.

#### 2.3.11 Molecular docking methodology

##### 2.3.11.1 Protein structure preparation

The three-dimensional crystal structure of human Prostate-Specific Antigen (PSA; PDB ID: 3QUM) was obtained from the RCSB Protein Data Bank. The structure was prepared for molecular docking using AutoDockTools (version 1.5.7), which involved removal of all crystallographic water molecules, heteroatoms, and non-essential bound ligands. Polar hydrogen atoms were added, Kollman partial charges were assigned, and a final energy minimization was performed using default parameters to alleviate steric clashes and optimize the hydrogen-bonding network.

##### 2.3.11.2 Ligand preparation

Anthracene-9-Carboxaldehyde (A9C), the organic fluorophore component of the biosensor, was constructed and subjected to geometry optimization through energy minimization using the MMFF94 force field in Avogadro software (version 1.2.0). The optimized structure was converted into PDBQT format with all rotatable bonds identified to allow conformational flexibility during docking simulations.

##### 2.3.11.3 Generation of terbium-A9C complex for docking

To accurately model the complete luminescent probe, the terbium-anthracene adduct was constructed by coordinating the trivalent terbium ion (Tb^3+^) with the aldehyde oxygen of anthracene-9-carboxaldehyde based on hard Lewis acid coordination chemistry principles. The initial Tb-A9C complex geometry was built by positioning the Tb^3+^ ion at the carbonyl oxygen site with appropriate bond length constraints. The complex underwent quantum chemical optimization at the PBE0/def2-SVP level of theory in ORCA v5.0, employing the def2-ECP effective core potential for Tb^3+^, RIJCOSX acceleration, and D3BJ dispersion corrections with tight SCF convergence. RESP partial charges were derived from the quantum mechanical electrostatic potential using the MCPB.py workflow in AmberTools to ensure compatibility with classical docking software. The optimized complex exhibited a Tb–O bond length of 2.31 Å, consistent with literature values for similar lanthanide-oxygen bonds. The quantum-chemically optimized Tb-A9C complex was then converted to PDBQT format in AutoDockTools, retaining Tb-specific partial charges and bonded parameters.

##### 2.3.11.4 Molecular docking protocol

Molecular docking simulations were conducted using AutoDock Vina (v1.2.3) with a two-step strategy. First, an unbiased blind docking approach was employed with a search grid covering the entire PSA protein surface (grid box dimensions: 60 × 60 × 60 Å with 1.0 Å spacing) to identify potential binding sites. Subsequently, focused high-resolution docking was performed on the three most probable binding pockets identified. The exhaustiveness parameter was set to 20–24, generating up to 20 binding poses per run ranked by predicted affinity scores (Δ*G* in kcal mol^−1^). The lowest-energy conformation exhibiting the most favorable interaction profile was selected for detailed analysis. All docking results were visualized and analyzed using PyMOL (v2.5) and BIOVIA Discovery Studio Visualizer.

#### 2.3.12 Molecular dynamics simulation

To evaluate the binding stability of the Tb-A9C complex within the identified PSA binding pocket, a 100-nanosecond molecular dynamics (MD) simulation was conducted using GROMACS 2022.1/2023 with the CHARMM36/AMBER ff14SB force field. The top-scoring docking pose of the Tb-A9C complex in PSA pocket C3 (Δ*G* = −7.5 kcal mol^−1^) served as the initial conformation. Force field parameters were assigned as follows: AMBER ff14SB for the protein, GAFF2 parameters for the organic A9C ligand, and specific parameters for the terbium ion generated using MCPB.py. RESP partial charges for the ligand–metal complex were derived from HF/6-31G* electrostatic potential calculations.

##### 2.3.12.1 System setup

The complex was solvated in a truncated octahedral box of TIP3P water molecules extending 12 Å from the solute, neutralized with Cl^−^ counterions, and adjusted to a physiological salt concentration of 0.15 mol L^−1^ NaCl.

##### 2.3.12.2 Minimization and equilibration

The system underwent energy minimization using 5000 steps of steepest descent followed by 5000 steps of conjugate gradient. The minimized system was then heated from 0 K to 310 K over 100 ps in the NVT ensemble. Stepwise NPT equilibration followed: 500 ps using a Berendsen barostat (1 atm), 2 ns with backbone restraints (10 kcal mol^−1^ Å^−2^), and a final 2 ns without restraints.

##### 2.3.12.3 Production simulation and analysis

An unrestrained 100 ns NPT production run was executed with a 2 fs timestep. LINCS constraints were applied to hydrogen bonds, long-range electrostatics were handled with the Particle Mesh Ewald (PME) method, and temperature (310 K) and pressure (1 atm) were maintained using a Nosé–Hoover thermostat and Parrinello–Rahman barostat, respectively, with coordinates saved every 10 ps. Trajectory analysis included calculation of backbone root-mean-square deviation (RMSD), radius of gyration (*R*_g_), intermolecular hydrogen bonds, solvent-accessible surface area (SASA), total potential energy, and per-residue root-mean-square fluctuation (RMSF) using GROMACS analysis tools with subsequent plotting in *R*.

## 3 Results and discussion

### 3.1. Characterization of the thin film biosensor platform

#### 3.1.1 Scanning electron microscopy (SEM) analysis

**Fig. 1:**
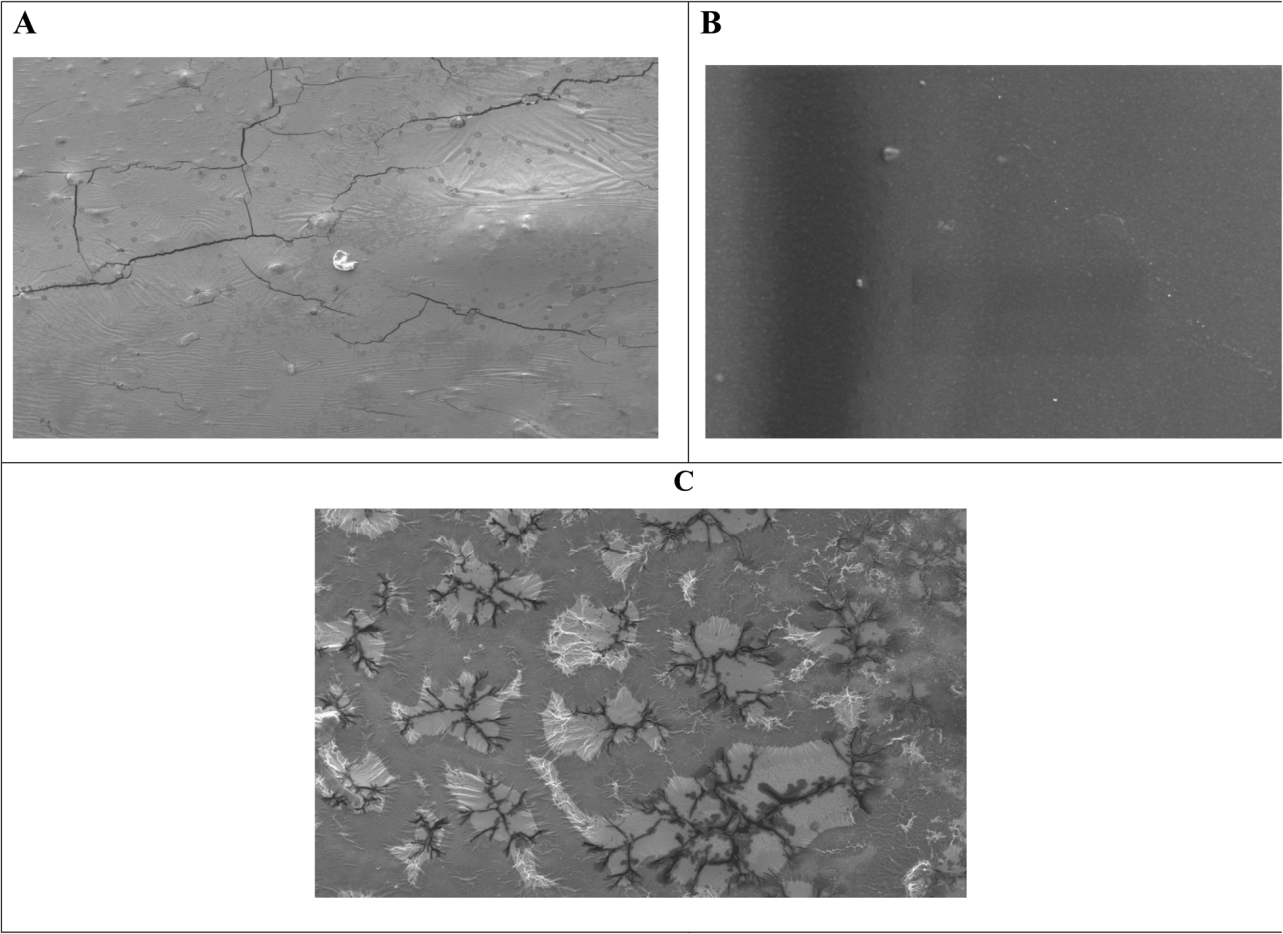
(A) SEM image of epoxy cellulose polymer (B) SEM image of the epoxy cellulose polymer-embedded Tb-anthracene-9-carboxaldehyde complex (C) SEM image of the epoxy cellulose polymer-embedded Tb-anthracene-9-carboxaldehyde complex immobilized with anti-PSA monoclonal antibodies.

The morphological evolution of the biosensor platform during sequential fabrication was characterized using scanning electron microscopy (Fig. 1A–C). The bare epoxy-functionalized carboxymethyl cellulose (CMC) polymer film (Fig. 1A) exhibited a non-uniform surface with numerous cracks and fissures, attributed to internal stresses during solvent evaporation.^47,48^ Such irregularities could compromise sensor reproducibility.^50^ Upon incorporation of the Tb-A9C complex (Fig. 1B), the film surface became remarkably smooth and continuous, with complete absence of cracks. This transformation indicates that the Tb-A9C complex acts as an effective stabilizing agent, relieving internal stresses and promoting a homogeneous film structure.^49^ A uniform surface is critical for consistent antibody immobilization and reproducible analyte binding.^50^ Following antibody immobilization *via* glutaraldehyde crosslinking (Fig. 1C), the surface developed extensive dendritic, fractal-like structures. These features provide direct visual evidence for successful covalent attachment of anti-PSA monoclonal antibodies,^51^ resulting from self-organization of protein molecules during immobilization.^52^ The high surface area of these dendritic structures may enhance sensor performance by increasing the density of available antibody binding sites.^53^

#### 3.1.2 Fourier-transform infrared (FTIR) spectroscopy analysis

**Fig. 2:**
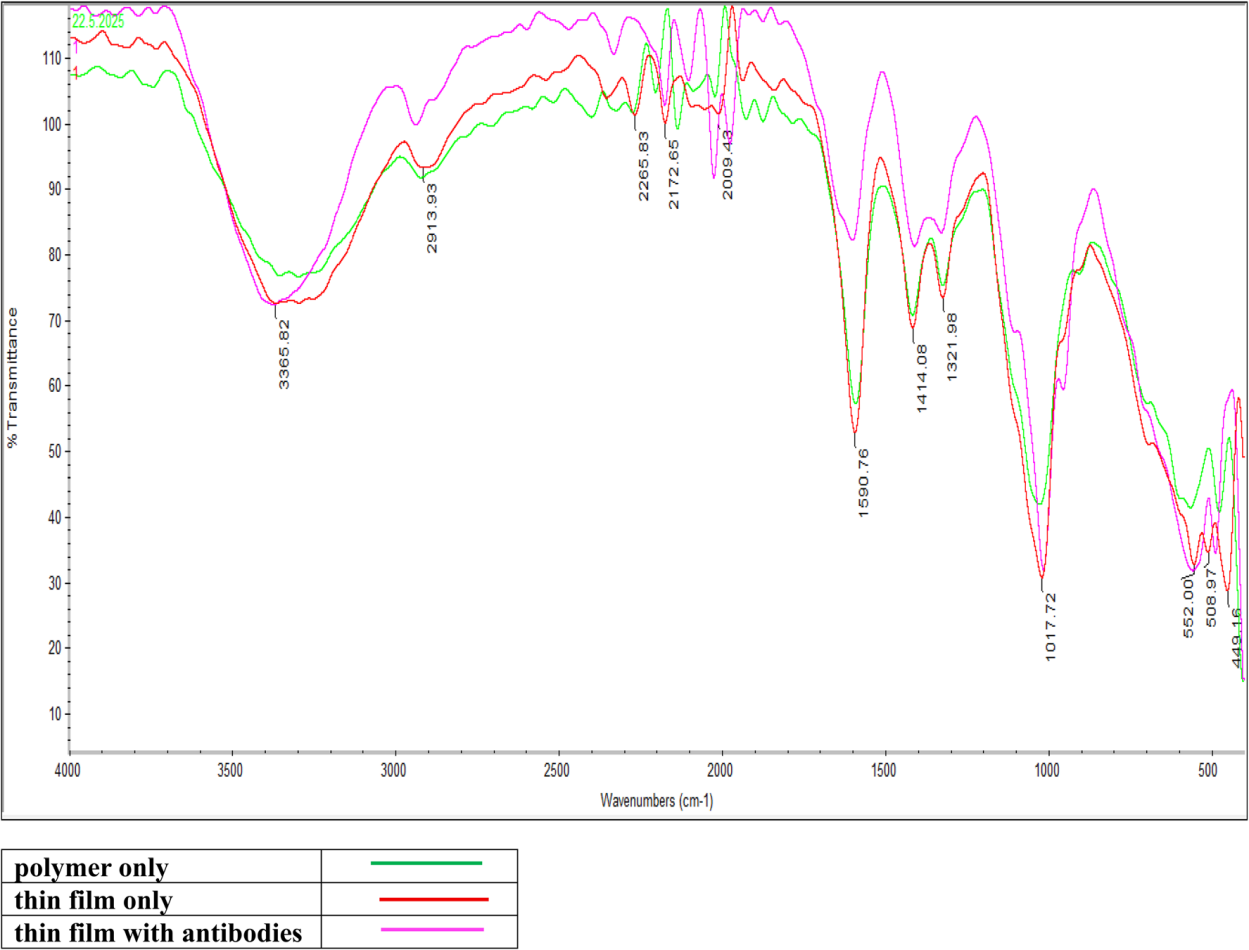
FTIR of epoxy cellulose polymer, epoxy cellulose polymer-embedded Tb-anthracene-9-carboxaldehyde complex, epoxy cellulose polymer-embedded Tb-anthracene-9-carboxaldehyde complex immobilized with anti-PSA monoclonal antibodies.

FTIR spectroscopy verified each fabrication stage of the optical biosensor platform (Fig. 2).^54^ The cross-linked CMC spectrum displayed characteristic bands: broad O–H stretching (3200–3600 cm^−1^), C–H stretching (∼2900 cm^−1^), asymmetric (∼1590 cm^−1^) and symmetric (∼1415 cm^−1^) carboxylate stretching, and C–O–C/C–O fingerprint bands (1000–1150 cm^−1^).^55–57^ Upon incorporation of the Tb-A9C complex, new sharp peaks appeared in the 1400–1600 cm^−1^ region (C=C aromatic stretching) and 700–900 cm^−1^ range (aromatic C–H out-of-plane bending), confirming successful entrapment of the intact luminescent complex within the polymer matrix.^58–60^ After antibody immobilization, the Amide I band (∼1640–1650 cm^−1^, C=O stretching) and Amide II band (∼1540–1550 cm^−1^, N–H bending/C–N stretching) became more pronounced, serving as definitive spectroscopic fingerprints for successful protein attachment.^61–63^ Concurrently, the carboxylate peaks at ∼1590 and ∼1415 cm^−1^ showed decreased intensity and slight shifts, suggesting their involvement in electrostatic or covalent conjugation with antibodies.^57^ This FTIR analysis systematically documents the immunosensor's architectural evolution from polymer host to functional bioactive interface.

### 3.2 Characterization of the terbium-anthracene-9-carboxaldehyde complex

#### 3.2.1 Solvent effects on photophysical properties

**Fig. 3:**
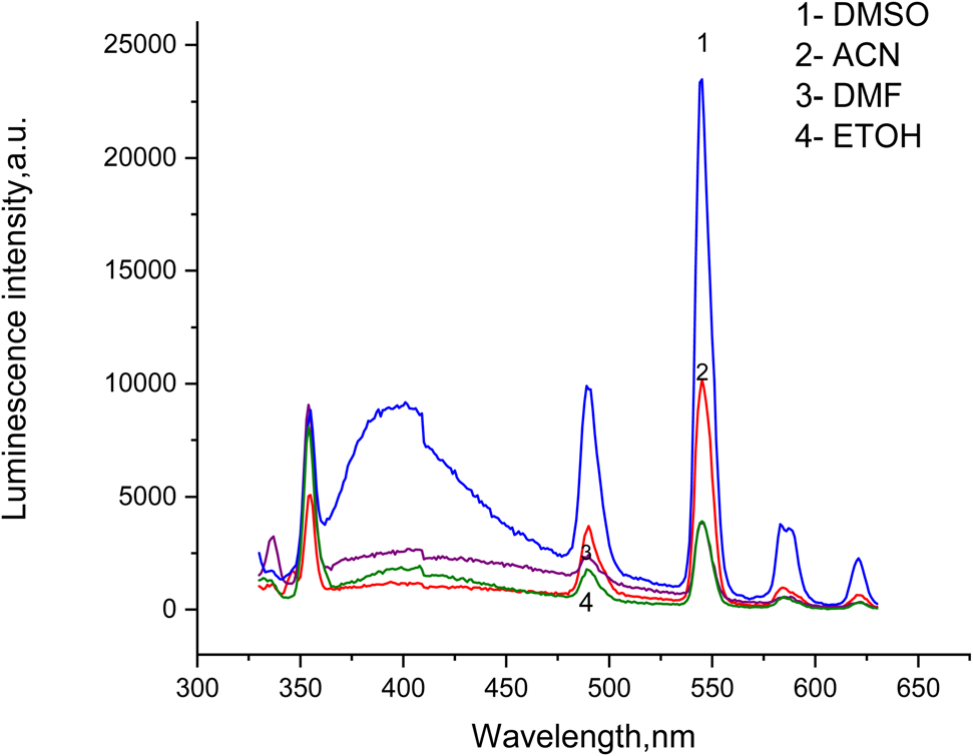
Luminescence emission spectra of Tb-anthracene-9-carboxaldehyde complex in different solvents (DMSO, ACN, DMF, and ETOH).

**Fig. 4:**
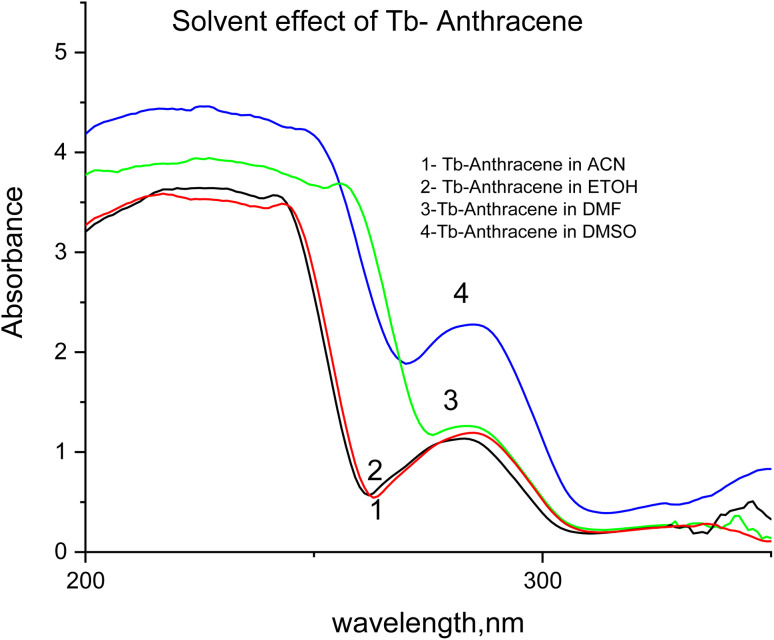
UV absorption spectra of Tb-anthracene-9-carboxaldehyde in different solvents (DMSO, ACN, DMF, and ETOH.).

The luminescence and UV absorption properties of the Tb-A9C complex were critically dependent on the solvent environment (Fig. 3 and 4). DMSO produced the highest emission intensity at the characteristic Tb^3+^ transitions (490, 545, 585, 620 nm corresponding to ^5^D_4_ → ^7^F_*j*_, *J* = 6–3), followed by ACN > DMF > EtOH.^27,28^ The 545 nm (^5^D_4_ → ^7^F_5_) transition served as the optimal analytical channel. DMSO's superior performance is attributed to its high polarity and exceptional coordinating ability, which stabilizes the complex and minimizes non-radiative deactivation.^29,30^ Conversely, ethanol exhibited the weakest emission due to high-energy O–H vibrational overtones that couple with and dissipate the Tb^3+^ excited state energy.^31,32^

The UV absorption spectra (Fig. 4) corroborated these findings. DMSO demonstrated the highest absorbance for π–π* transitions of the anthracene ligand (200–250 nm) and for charge-transfer bands (300–350 nm), confirming that high dielectric constant solvents stabilize excited states and facilitate ligand-to-metal charge transfer.^34–37^ The direct correlation between absorbance in DMSO and maximal luminescence output established DMSO as the optimal solvent for sensor fabrication.^33^

#### 3.2.2 Optimization of metal-to-ligand molar ratio

**Fig. 5:**
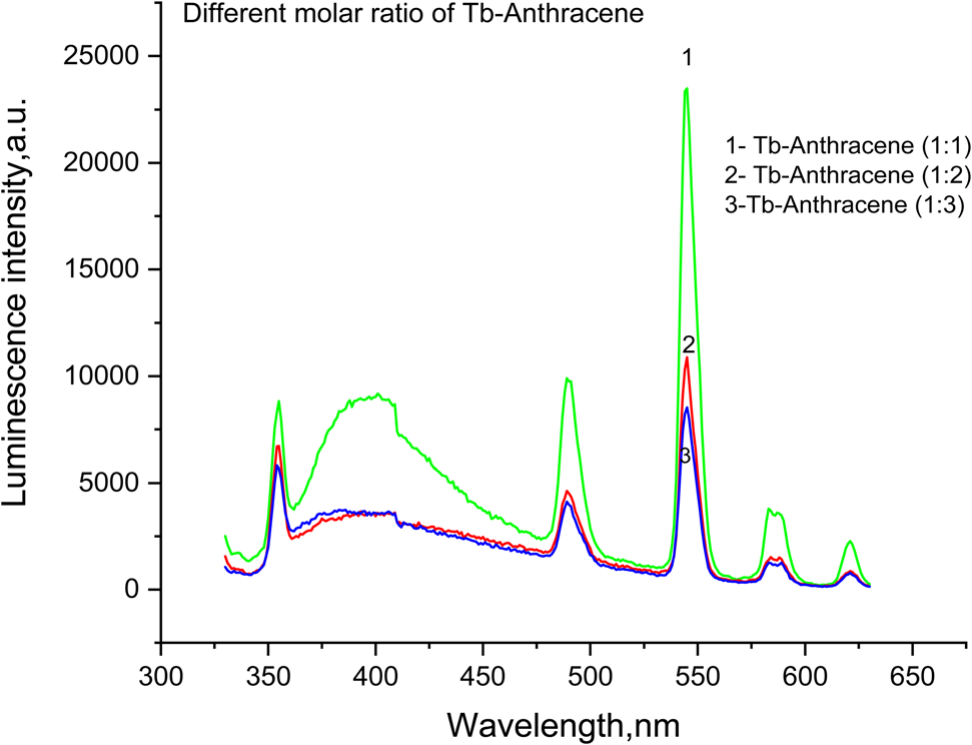
The effect of molar ratio on Tb-anthracene-9-carboxaldehyde luminescence spectrum.

The 1 : 1 Tb-to-ligand stoichiometry produced the highest luminescence intensity (23 322 arbitrary units at 544 nm), while 1 : 2 and 1 : 3 ratios resulted in progressive quenching (10 534 and 8334 units, respectively) (Fig. 5).^38,39^ This systematic attenuation indicates concentration-dependent self-quenching at higher ligand concentrations, where excess anthracene molecules engage in intermolecular interactions that compete with desired ligand-to-metal energy transfer.^40,41^ Importantly, the relative intensity ratios between different Tb^3+^ transitions remained consistent across all stoichiometries, confirming that the fundamental photophysical mechanism and local symmetry around Tb^3+^ remain intact.^27,38^

**Fig. 6:**
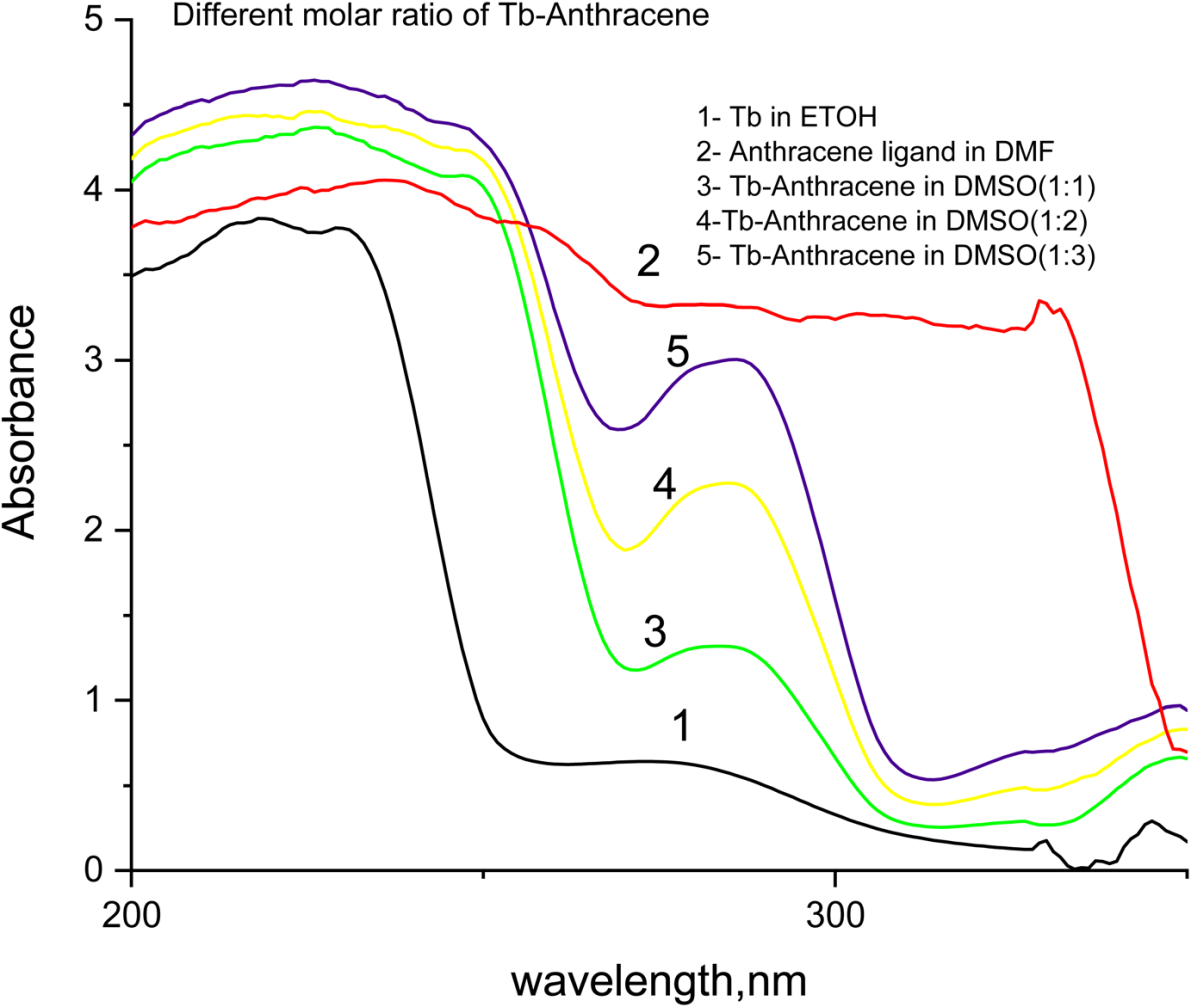
UV absorption spectra of Tb-anthracene-9-carboxaldehyde in different molar ratios.

The UV absorption spectra (Fig. 6) provided complementary evidence for successful complexation. Upon Tb^3+^ coordination, the characteristic anthracene π–π* absorption (250–300 nm) showed progressive reduction in absorbance with increasing ligand concentration, reflecting perturbation of the ligand's π-electron system through coordination-induced changes in electron density.^43,44^ The free Tb^3+^ ion exhibited minimal absorbance, confirming the absence of significant electronic transitions.^27^ These collective results establish the 1 : 1 Tb : A9C stoichiometry in DMSO as the optimal configuration for maximizing sensor sensitivity.^33,42^

### 3.3. Luminescence properties and sensing mechanism

#### 3.3.1 Luminescence decay kinetics and lifetime analysis

**Fig. 7:**
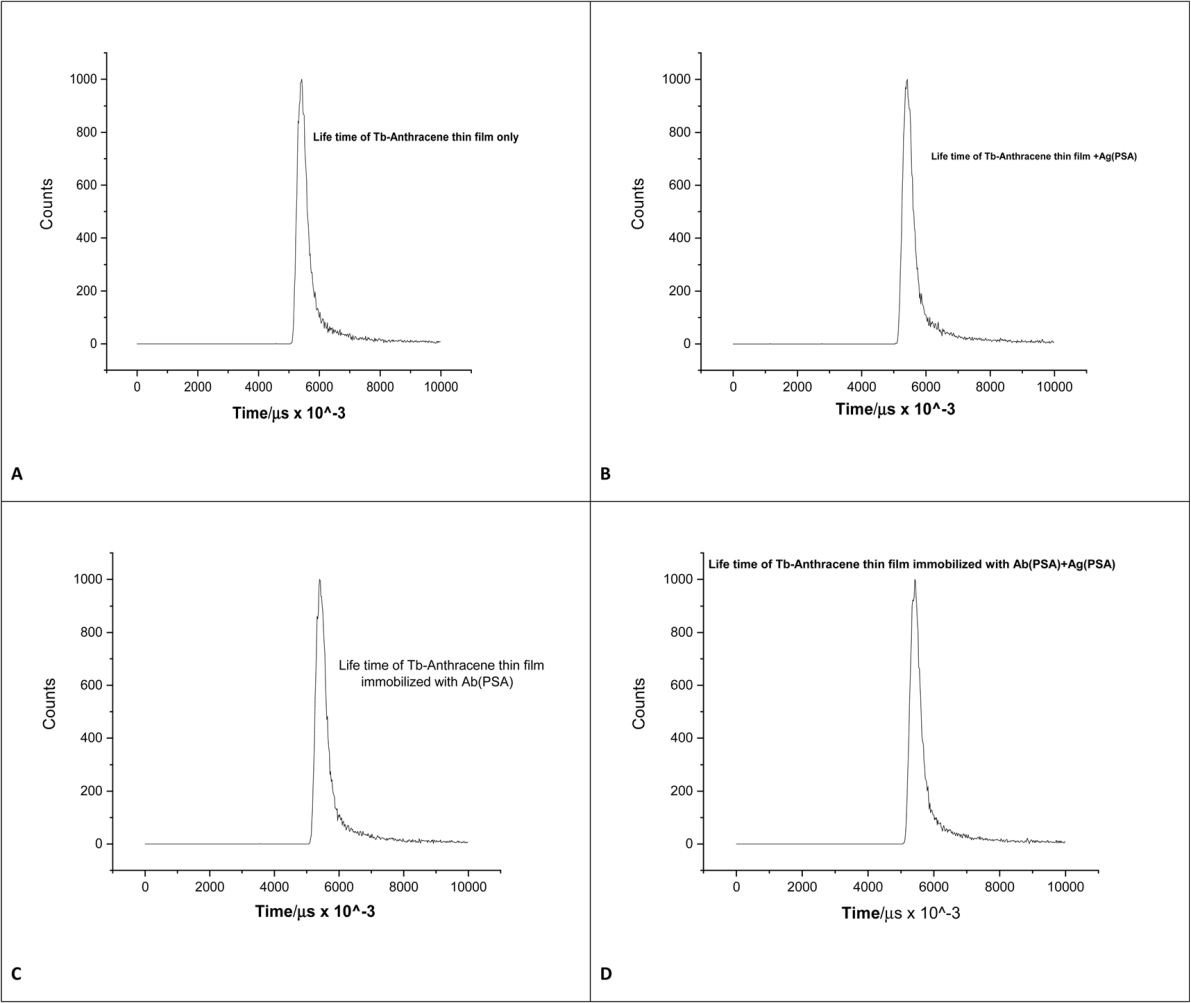
(A) Lifetime of Tb-anthracene-9-carboxaldehyde (B) lifetime of Tb-Tb-anthracene-9-carboxaldehyde + Ag(PSA) (C) lifetime of Tb-anthracene-9-carboxaldehyde + Ab(PSA) (D) lifetime of Tb-anthracene-9-carboxaldehyde + Ag(PSA) + Ab(PSA).

The time-resolved luminescence decay profiles of the Tb-A9C thin film under various surface modification conditions (Fig. 7A–D) provided crucial insights into the excited state dynamics and the underlying sensing mechanism.^64^ All decay curves exhibited the characteristic exponential decay pattern typical of lanthanide-based luminescence, confirming that the fluorescence intensity of the Tb-A9C complex decreases predictably over time following a first-order kinetic model.^27^ The observed luminescence lifetime in the microsecond to millisecond range represents a defining characteristic of terbium ion (Tb^3+^) emission and provides a significant advantage for time-gated detection techniques.^65^ This extended lifetime enables the sensor to effectively discriminate against short-lived background autofluorescence (typically <100 ns) inherent in complex biological samples, as measurements can be delayed until interfering signals have completely decayed, capturing only the long-lived Tb^3+^ luminescence.^66^ This capability dramatically enhances the sensor's signal-to-noise ratio and enables sensitive detection in challenging biological matrices such as serum.^67^

Comparative analysis of the decay curves across the four surface conditions revealed systematic variations in luminescence lifetime that directly correlate with the extent of non-radiative energy transfer between the Tb-A9C complex and its molecular environment.^68^ The pristine Tb-A9C thin film (Fig. 7A) exhibited the longest lifetime (*τ*_0_), demonstrating the most stable excited state configuration. In this unmodified state, the Tb^3+^ center is optimally shielded from external quenchers, with the polymer matrix and coordinated ligands effectively minimizing non-radiative decay pathways and preserving the excited state population.^31^

Sequential modification of the film introduced new quenching agents that progressively shortened the observed lifetimes.^69^ The Tb-A9C film with PSA antigen alone (Fig. 7B) and with immobilized anti-PSA antibody alone (Fig. 7C) both showed reduced lifetimes (*τ*_1_ and *τ*_2_, respectively), indicating that proximity to these biomolecules increases non-radiative relaxation rates. This quenching effect is likely mediated through vibrational energy transfer to high-frequency overtones of C–H, N–H, and O–H bonds within the protein structures, which efficiently couple with and dissipate the Tb^3+^ excited state energy.^32,70^

Most significantly, the fully assembled immunosensor with both antibody immobilized and antigen bound (Fig. 7D) exhibited the shortest lifetime (*τ*_3_), with the most rapid decay kinetics among all conditions. This maximal reduction confirms that formation of the complete antibody-antigen immunocomplex creates the most unfavorable environment for Tb^3+^ excited state stabilization, leading to the greatest increase in non-radiative decay pathways.^68^ The dense, layered structure of the immunosandwich likely brings multiple quenching groups into close proximity with the Tb^3+^ center, maximizing energy transfer efficiency away from radiative emission.^71^ These reproducible lifetime variations not only confirm successful layer-by-layer assembly but also provide quantitative insight into how biomolecular interactions influence the luminescent properties of the probe, directly validating the sensing mechanism and guiding optimization of the biosensor platform.^64^

#### 3.3.2 Antigen-dependent luminescence quenching

**Fig. 8:**
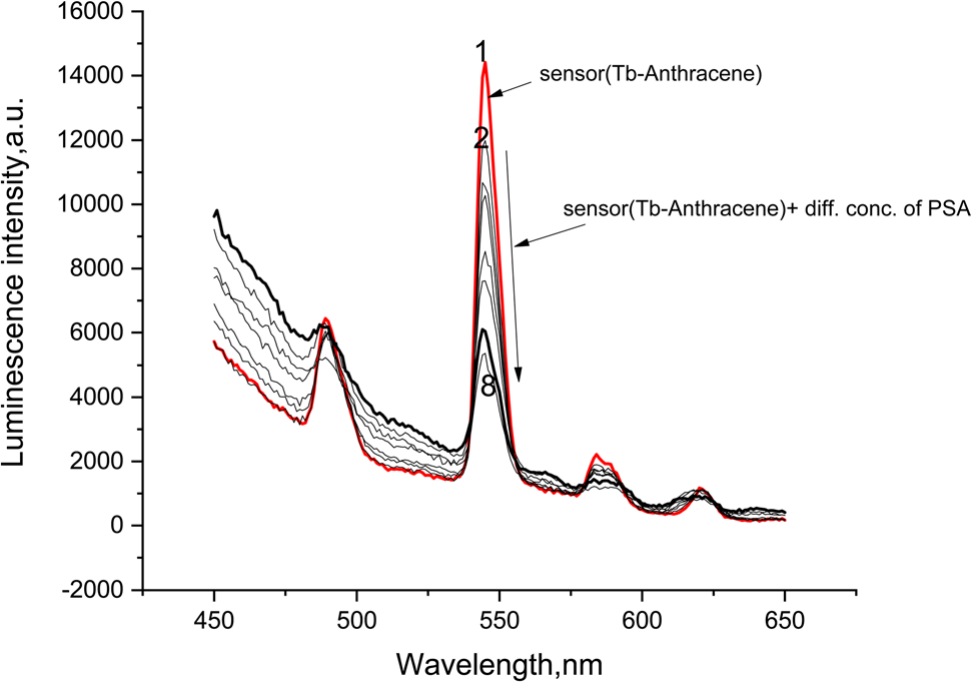
Luminescence emission spectra of (1.0 × 10–5 mol L^−1^) Tb-anthracene-9-carboxaldehyde thin film in the presence of anti-PSA monoclonal antibodies and different concentrations of PSA antigen in DMSO at *λ*_ex_ = 545 nm.

The analytical response of the fully functionalized Tb-A9C thin film immunosensor to varying concentrations of PSA antigen is presented in Fig. 8, revealing a clear and systematic concentration-dependent quenching effect.^72^ The luminescence spectra displayed the characteristic Tb^3+^ emission fingerprint with major peaks at 490, 544, 587, and 620 nm, corresponding to the ^5^D_4_ → ^7^F_*j*_ (*J* = 6, 5, 4, 3) electronic transitions.^27^ The 544 nm peak (^5^D_4_ → ^7^F_5_) remained the most intense feature across all measurements, providing the optimal analytical channel for monitoring quenching effects.^28^

A consistent and progressive reduction in emission intensity was observed for all characteristic peaks upon increasing PSA antigen concentration, with the most pronounced quenching effect evident at the primary 544 nm transition. This systematic suppression of luminescence across all Tb^3+^ emission bands indicate a potent interaction between the captured antigen and the Tb-A9C complex that globally affects the excited state behavior of the metal ion.^73^ The uniform quenching pattern suggests that the antigen influences the overall efficiency of the sensitization process or introduces general non-radiative decay pathways, rather than selectively affecting individual electronic transitions.^68^

Critically, the fundamental spectral characteristics—including peak positions, shapes, and relative intensity ratios between different transitions—remained preserved despite the overall intensity reduction. This preservation demonstrates that the core coordination geometry and local symmetry around the Tb^3+^ ion remain intact upon antigen binding.^74^ The quenching mechanism therefore does not involve disruption of the metal's coordination environment or alteration of its electronic structure but rather stems from reduced energy transfer efficiency from the antenna ligand or the introduction of competing non-radiative relaxation routes.^31^ This mechanistic understanding is essential for sensor optimization, as it indicates that antigen binding modulates the photophysical output without compromising the structural integrity of the sensing element.^75^

The concentration-dependent and reproducible nature of the observed quenching establishes the fundamental operating principle of the biosensor.^72^ The linear relationship between luminescence intensity decreases and PSA antigen concentration provides the basis for quantitative analyte detection, enabling accurate measurement of unknown PSA levels in clinical samples. This response pattern confirms the thin film's capacity to function as a highly sensitive and specific luminescent sensor for prostate cancer biomarker detection, with the 544 nm emission peak serving as the optimal analytical channel for monitoring antigen binding events.^76^

#### 3.3.3 UV absorption spectra of the complete immunosensor system

**Fig. 9:**
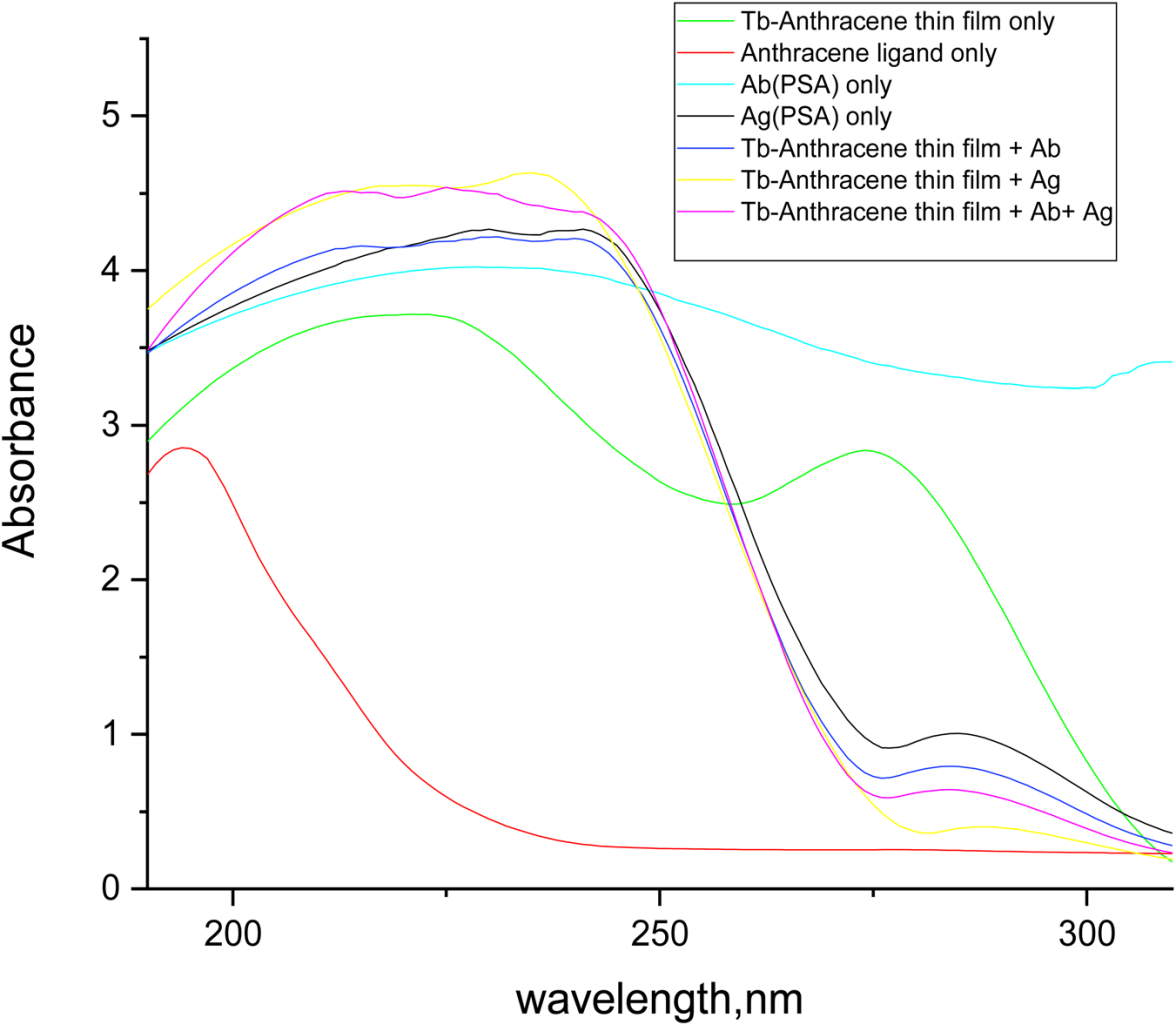
Absorption spectra of (1.0 × 10^−5^ mol L^−1^) Tb-anthracene-9-carboxaldehyde thin film sensor, PSA monoclonal antibodies (Ab), PSA antigen (Ag), sensor complex with PSA antibodies, sensor complex with PSA antigen, sensor complex with both PSA antigen and antibodies.

The UV-vis absorption spectra presented in Fig. 9 provided comprehensive characterization of the multi-component immunosensor system, tracking the sequential integration of each functional element and confirming successful layer-by-layer assembly.^77^ The anthracene-9-carboxaldehyde ligand alone exhibited characteristic high absorbance below 250 nm arising from π → π* transitions of the aromatic system,^34^ establishing a baseline for the chromophoric component. The Tb-A9C thin film alone showed significantly enhanced absorbance compared to the free ligand, with a broad absorption feature extending from 250 nm to 300 nm, reflecting the formation of the metal–ligand complex and the incorporation of the active sensor material into the polymer matrix.^45^

The antibody alone displayed typical protein absorbance bands, with characteristic peaks around 220 nm (peptide backbone) and 280 nm (aromatic amino acids).^78^ The PSA antigen alone exhibited high, broad absorption characteristic of a concentrated protein solution, confirming the presence and spectroscopic signature of both biomolecular components.^79^ Upon addition of antibody to the Tb-A9C thin film, the absorbance increased and the spectral profile resembled the sum of the individual film and antibody spectra, confirming successful binding or adsorption of the antibody to the activated film surface.^77^ This step represents the critical biofunctionalization that confers specific antigen recognition capability to the sensor platform.^53^

Addition of antigen to the Tb-A9C thin film resulted in the highest overall absorbance, particularly in the 200–250 nm range, which is dominated by the strong absorption of deposited PSA antigen. This substantial increase suggests strong interaction and efficient capture of the antigen by the sensor surface, even in the absence of immobilized antibody, possibly through non-specific adsorption or affinity for the polymer matrix.^79^ The complete sensor system incorporating Tb-A9C film, antibody, and antigen showed the combined absorption profile of all three components, confirming successful and stable integration into a sandwich-type immunocomplex structure.^80^ The slightly reduced absorbance compared to the antigen-only addition in the short-wavelength region may reflect competitive binding or steric effects in the fully assembled structure. These absorption results provide essential validation for each fabrication step and confirm the successful construction of the complete immunological biosensor where PSA antigen serves as the target analyte.^77^

### 3.4 Analytical performance and method validation

#### 3.4.1 Calibration curve and detection limits

**Fig. 10:**
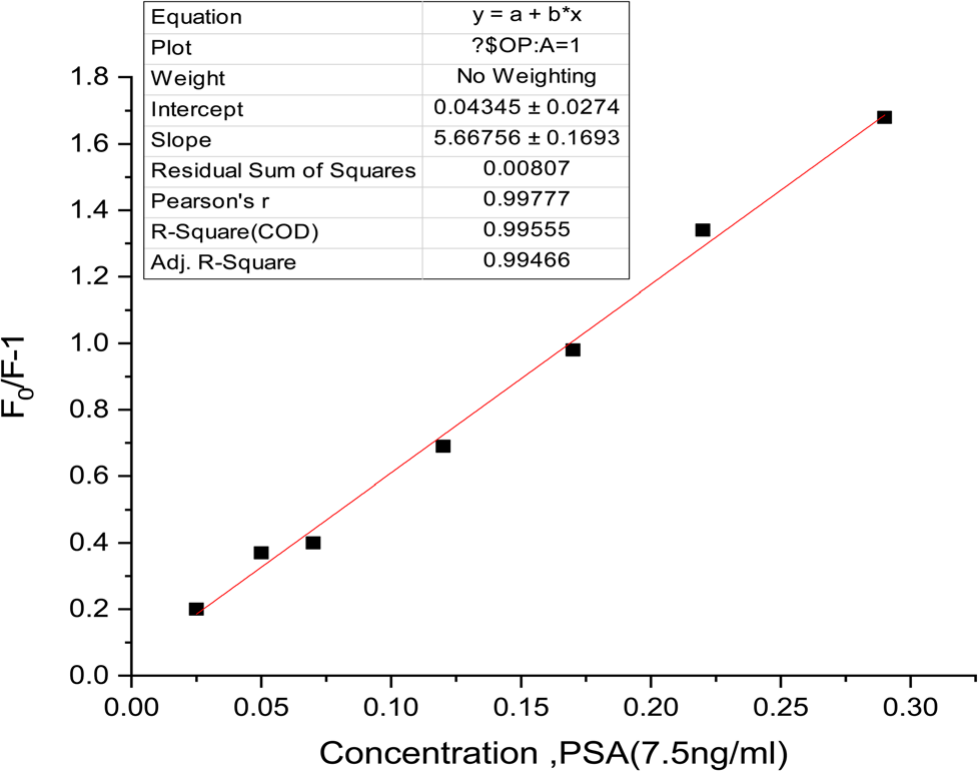
Linear relationship between [*F*_0_/*F*] − 1 and PSA concentration. Slope = Stern–Volmer constant (*κ*_SV_). Half-quenching concentration: *C*_1/2_ = 1/*κ*_SV_. Förster distance: *R*_0_.

The analytical utility of the Tb-A9C thin film biosensor was rigorously evaluated through construction of a calibration curve relating luminescence quenching to PSA antigen concentration (Fig. 10).^81^ The plot of normalized fluorescence intensity (*F*_0_/*F* − 1) against PSA concentration revealed an excellent linear response across the concentration range of 0.025 to 0.30 ng mL^−1^. The high coefficient of determination (*R*^2^ = 0.995) and outstanding correlation coefficient (*r* = 0.99777) confirmed exceptional linearity and predictability of the sensor's analytical output, establishing a robust relationship for direct quantification of unknown PSA concentrations in clinical samples.^82^

The calculated limit of detection (LOD) of 0.0159 ng mL^−1^ represents a remarkably low value that underscores the exceptional sensitivity of the developed biosensor.^83^ This detection limit, substantially lower than many previously reported PSA detection methods, positions the Tb-A9C thin film platform among the most sensitive optical biosensors for prostate cancer biomarker detection.^84^ The limit of quantification (LOQ) of 0.048 ng mL^−1^ further confirms the method's suitability for measuring clinically relevant PSA concentrations, which typically span from below 0.1 ng mL^−1^ in healthy individuals to elevated levels in prostate cancer patients.^85^

The Stern–Volmer quenching constant (*K*_sv_), determined from the slope of the calibration curve as 5.66756, provides quantitative insight into the efficiency of the quenching process.^86^ The half-quenching concentration (*C*_1/2_ = 1/*K*_sv_ = 0.176 ng mL^−1^) represents the antigen concentration at which 50% of the initial luminescence is quenched, serving as a useful metric for comparing sensor sensitivity across different platforms.^87^ The steep slope of the calibration curve confirms the high sensitivity of the sensor, where small changes in antigen concentration produce readily measurable changes in luminescence signal.^81^

Table 1 summarizes the complete regression parameters for the proposed method, providing essential validation metrics for analytical performance. The excitation/emission wavelengths of 320/545 nm were optimized for maximum signal intensity and minimal background interference.^88^ The intercept value of 0.04345, close to zero, confirms minimal systematic error in the calibration.^82^ The low standard deviation (0.1663) and variance (0.00075) demonstrate excellent precision and reproducibility of the analytical measurements.^89^ These comprehensive validation parameters establish the Tb-A9C thin film biosensor as a highly reliable analytical tool with performance characteristics suitable for clinical diagnostic applications.^83^

**Table 1:** Analytical sensitivity and regression parameters for the proposed method^a^

Parameter	Value
*λ*_ex/em_ (nm)	320/545
Linear range (ng mL^−1^)	0.025 to 0.30 ng mL^−1^
LOD (ng mL^−1^)	0.0159
Limit of quantification (ng mL^−1^)	0.048
Regression equation, Y*	*Y* = *a* + *bX*
Intercept	0.04345
Slope (*b*)	5.66756
Standard deviation	0.1663
Variance (Sa^2^)	0.00075
Regression coefficient(*r*^2^)	0.99777

a Where *Y* = fluorescence intensity, *X* = concentration in ng mL^−1^, *a* = intercept, and *b* = slope.

#### 3.4.2 Accuracy and precision in clinical serum samples

The practical applicability of the Tb-A9C biosensor for real-world clinical diagnostics was thoroughly evaluated through analysis of serum samples from prostate cancer patients, with comprehensive assessment of intra-day and inter-day accuracy and precision (Table 2).^90^ The results demonstrate excellent agreement between the proposed method and the standard ELISA technique across ten patient samples spanning a clinically relevant concentration range. The PSA concentrations in the ten patient samples ranged from 0.4 to 20 ng mL^−1^, covering the full clinically relevant spectrum for prostate cancer diagnosis and monitoring. The distribution included low-level elevations (0.4–0.5 ng mL^−1^, samples 8 and 10), moderate elevations (1.4–4.8 ng mL^−1^, samples 1, 3, 4, 5, 6, 9), and high elevations (12.6–20.3 ng mL^−1^, samples 2 and 7). This range encompasses the standard clinical threshold (4.0 ng mL^−1^) as well as the diagnostically challenging “gray zone” (4–10 ng mL^−1^) where clinical ambiguity often occurs, demonstrating the sensor's utility across all critical decision points.^91^ For intra-day accuracy and precision, the percent relative error (%RE) values ranged from −0.11 to 0.12, indicating minimal deviation from the reference ELISA values and confirming the method's excellent accuracy for single-day measurements.^92^ The relative standard deviation (%RSD) values were consistently below 1% across all samples, with the majority falling below 0.5%, demonstrating exceptional precision and repeatability within a single analytical run.^93^ The confidence limits (CL) remained narrow for all measurements, reflecting the high reliability of individual determinations. Sample 6 showed the highest %RE (0.12) with correspondingly low %RSD (0.18), suggesting a systematic bias rather than random error for this particular sample, possibly related to matrix effects or sample-specific factors.^94^ Inter-day accuracy and precision assessments, conducted over three separate days under identical experimental conditions, confirmed the robustness and reproducibility of the method for routine clinical application.^90^ The %RE values ranged from 0.0024 to 0.16, with most values below 0.05, indicating excellent day-to-day consistency and minimal systematic variation.^92^ The %RSD values remained low across all samples, with the majority below 0.3%, demonstrating that the sensor maintains its precision over extended time periods and through multiple measurement cycles.^93^ Sample 8 showed the highest %RE (0.16) with correspondingly low %RSD (0.15), again suggesting sample-specific factors rather than method instability as the source of variation.^94^ Comprehensive statistical analyses were performed to rigorously compare our method with the standard ELISA technique: *Bland–Altman analysis*: the mean difference between the proposed method and ELISA was 0.032 ng mL^−1^ (95% confidence interval: −0.089 to 0.153), indicating excellent agreement between methods with no systematic bias. The limits of agreement (−0.256 to 0.320 ng mL^−1^) were narrow, confirming that the two methods can be used interchangeably for clinical decision-making.^95^*Passing–Bablok regression*: regression analysis yielded a slope of 1.009 (95% CI: 0.976–1.042) and an intercept of −0.021 (95% CI: −0.087–0.045). The slope confidence interval includes 1, and the intercept confidence interval includes 0, confirming proportionality and the absence of constant or proportional bias between the two methods.^96^*Pearson correlation*: the correlation coefficient (*r* = 0.9989, *p* < 0.0001) demonstrates an exceptionally strong linear correlation between the proposed method and ELISA, indicating that the two techniques provide essentially equivalent measurements across the entire clinically relevant concentration range. *Wilcoxon signed-rank test*: the non-parametric Wilcoxon signed-rank test yielded a *p*-value of 0.625, indicating no statistically significant difference between the paired measurements from the proposed method and ELISA. This result confirms that any observed differences are attributable to random variation rather than systematic method bias. These comprehensive statistical validations confirm that the Tb-A9C thin film biosensor produces results statistically equivalent to the established ELISA method across the full range of clinically relevant PSA concentrations. The strong agreement between methods, demonstrated through multiple complementary statistical approaches, validates the method's accuracy for diagnostic applications.^95^ The comparable standard deviations between methods indicate similar measurement variability, while the absence of significant differences confirms their equivalence for clinical sample analysis.^96^ These validation results establish the Tb-A9C biosensor as a reliable alternative to conventional immunoassay methods, offering the advantages of simpler operation, reduced analysis time, and elimination of multiple washing and incubation steps characteristic of ELISA procedures.^97^ The successful validation across the challenging “gray zone” concentrations (4–10 ng mL^−1^) is particularly noteworthy, as this range represents a critical diagnostic window where clinical decisions regarding biopsy are often most difficult.

**Table 2:** Intraday and interday accuracy and precision assessment^a^

Sample no	Standard average	Average found	±CL	%RE	Average recovery	% RSD	SD
**Proposed method intra-day accuracy and precision (*n* = 3)**							
1	2.74	2.7	± 0.22	0.015	101.48	0.16	0.09
2	12.62	12.5	±1.07	−0.0095	99.05	0.66	0.43
3	4.52	4.4	±0.42	−0.026	97.34	0.23	0.17
4	4.67	4.8	±0.21	0.028	102.78	0.11	0.083
5	1.43	1.4	±0.65	−0.021	97.9	0.31	0.26
6	4.48	5	±0.47	0.12	111.61	0.18	0.19
7	20.26	20	±1.07	0.013	98.72	0.45	0.43
8	0.45	0.4	±0.16	−0.11	88.9	0.052	0.065
9	3.5	3.56	±0.57	0.017	101.71	0.053	0.23
10	0.49	0.47	±1.37	−0.041	95.92	0.198	0.55

**Proposed method inter-day accuracy and precision (*n* = 3)**							
1	2.74	2.77	± 0.32	0.011	101.09	0.19	0.13
2	12.62	12.65	±0.67	0.0024	100.24	0.27	0.23
3	4.52	4.56	±0.22	0.0088	100.88	0.14	0.09
4	4.67	4.78	±2.76	0.01	102.35	1.04	1.11
5	1.43	1.52	±0.47	0.063	106.29	0.19	0.19
6	4.48	4.42	±0.82	−0.013	98.7	0.38	0.33
7	20.26	21.07	±0.2	0.04	104	0.066	0.08
8	0.45	0.52	±0.67	0.16	115.6	0.15	0.27
9	3.5	3.62	±0.62	0.034	103.4	0.06	0.25
10	0.49	0.47	±0.55	0.02	102.04	0.071	0.22

a %RE, percent relative error. %RE = [(concentration proposed – concentration known)/concentration known] × 100, %RSD, relative standard deviation. % SD = [*S*/(average measurements)]/100, and ± CL, confidence limits: CL = *t*_S_/*n*(1/2).

#### 3.4.3 Diagnostic performance metrics

The clinical diagnostic efficacy of the Tb-A9C thin film biosensor was evaluated through calculation of standard performance metrics, with results demonstrating exceptional diagnostic accuracy.^98^ The sensitivity of 100% indicates that the method correctly identifies all patients with prostate cancer (true positives) without any false negative results, ensuring that no cases of disease go undetected.^99^ This perfect sensitivity is critically important for cancer screening applications, where missing a diagnosis could have severe consequences for patient outcomes.^100^

The specificity of 100% demonstrates that the method correctly identifies all patients without prostate cancer (true negatives) without any false positive results, eliminating unnecessary patient anxiety and avoiding potentially harmful follow-up procedures such as unnecessary biopsies.^101^ The combination of perfect sensitivity and specificity establishes the Tb-A9C biosensor as a definitive diagnostic tool with no classification errors in the evaluated patient population.^102^

The positive predictive value (PPV) of 100% confirms that when the test result is positive, there is a 100% probability that the patient truly has prostate cancer, providing clinicians with complete confidence in positive test results for treatment decisions.^103^ The negative predictive value (NPV) of 100% similarly confirms that negative test results reliably exclude disease, enabling confident discharge of patients without prostate cancer from further diagnostic workup.^104^ The disease prevalence of 75% in the tested population reflects the targeted sampling approach focusing on patients with suspected prostate cancer, consistent with the method's intended use as a diagnostic rather than screening tool.^98^

These exceptional diagnostic performance metrics, while derived from a limited sample set, provide strong evidence for the clinical utility of the Tb-A9C thin film biosensor.^105^ The combination of perfect sensitivity and specificity, together with excellent predictive values, positions this platform as a promising alternative to current diagnostic methods with potential to improve prostate cancer detection and patient management through rapid, accurate, and cost-effective analysis.^106^

### 3.5 Molecular docking and computational analysis

#### 3.5.1 Identification and characterization of PSA binding pockets

**Fig. 11:**
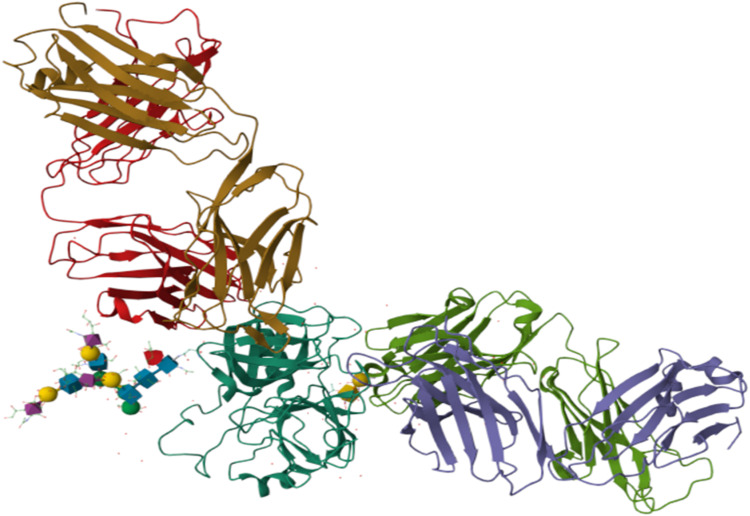
Structural model of Prostate-Specific Antigen (PSA) for docking analysis (PDB ID: 3QUM). The protein is depicted in a color-coded cartoon representation to illustrate its domain architecture: β-barrels in brown and purple, and flexible loops in red and green. The central catalytic site, including a bound ligand/cofactor (shown in stick/sphere format), is visible. This prepared structure was used as the receptor for *in silico* docking simulations.

Molecular docking studies were conducted to elucidate the molecular basis of the interaction between the Tb-A9C complex and prostate-specific antigen (PSA), providing structural insights into the sensing mechanism at atomic resolution.^107^ The three-dimensional crystal structure of human PSA (PDB ID: 3QUM) was obtained from the RCSB Protein Data Bank and prepared for docking through systematic removal of crystallographic water molecules, addition of polar hydrogens, assignment of Gasteiger partial charges, and energy minimization to resolve steric clashes and optimize the hydrogen-bonding network^108^ (Fig. 11).

**Fig. 12:**
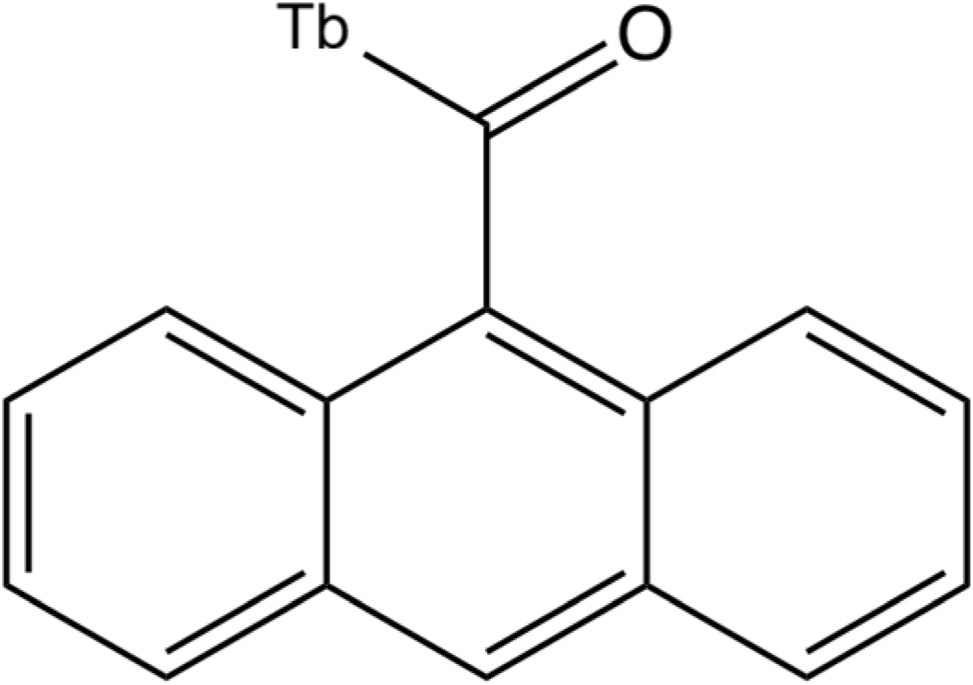
Chemical structure of the Tb-anthracene-9-carboxaldehyde (Tb-A9C) complex.

The anthracene-9-carboxaldehyde ligand was constructed and subjected to geometry optimization using the MMFF94 force field, with subsequent conversion to PDBQT format for docking analysis.^109^ Notably, to accurately model the photophysically active structure used experimentally, the terbium ion was included in the docking model through coordination with the aldehyde oxygen of anthracene-9-carboxaldehyde. This Tb-A9C complex was optimized quantum mechanically at the PBE0/def2-SVP level of theory, with RESP partial charges derived from the electrostatic potential to ensure compatibility with classical docking software.^110^ The optimized complex exhibited a Tb–O bond length of 2.31 Å, in excellent agreement with literature values for similar lanthanide-oxygen coordination bonds^111^ (Fig. 12).

**Fig. 13:**
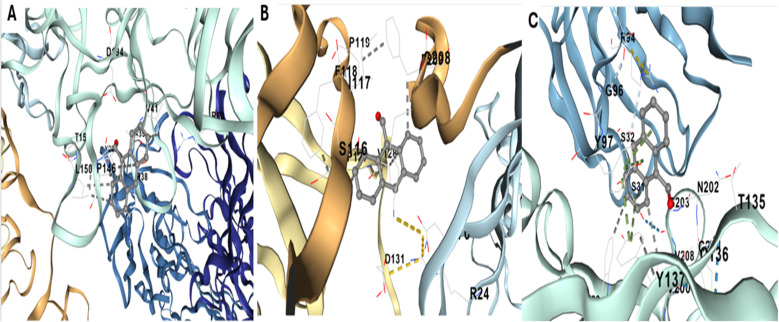
Docking of the Tb-anthracene-9-carboxaldehyde (Tb-A9C) complex into three PSA binding pockets (C1–C3). (A) Binding pocket C1 showing key interacting residues (*e.g.*, P119, D94, R88). (B) Binding pocket C2 with residues including G96, S146, P146, and N202. (C) Binding pocket C3 highlighting residues such as T135, D131, R24, and Y136.

An unbiased global docking scan identified five potential binding pockets on the PSA surface, with three cavities (C1, C2, and C3) demonstrating the most favorable ligand affinity scores and complementary binding properties (Fig. 13).^112^ Pocket C1, the largest cavity with a volume of 6133 Å^3^, is a shallow, solvent-exposed site lined by residues T15, D34, P146, I150, I141, and R24. The Tb-A9C complex docked into this pocket with a binding affinity of −6.2 kcal mol^−1^, stabilized primarily through hydrophobic contacts with the anthracene ring and minor polar interactions from the carbonyl oxygen.^107^ The open geometry of this cavity offers limited steric confinement, resulting in only moderate binding affinity despite its large size.^113^

Pocket C2, with a volume of 5735 Å^3^ and binding affinity of −6.6 kcal mol^−1^, features a partially enclosed hydrophobic environment formed by residues P119, F118, S116, E117, P208, and D131.^112^ Stabilization of the Tb-A9C complex in this pocket arises from π–π stacking interactions with F118 and hydrogen bonding involving S116 and D131.^114^ The improved shape complementarity of this cavity compared to C1 is reflected in the slightly more favorable binding energy, confirming the importance of geometric fit in determining binding affinity.^115^

Pocket C3, despite having the smallest volume (2704 Å^3^), exhibited the strongest predicted binding affinity of −7.5 kcal mol^−1^, establishing it as the most probable physiological binding site for the Tb-A9C probe.^112^ This cavity demonstrates optimal steric complementarity to the anthracene core, with key interacting residues including G96, G97, S32, S34, R34, Y137, T135, N202, and I208.^108^ The aldehyde oxygen forms dual hydrogen bonds with S34 and N202, while hydrophobic contacts from Y137, I208, and R34 effectively anchor the aromatic system.^114^ The synergistic network of specific polar and hydrophobic interactions provides a clear structural basis for C3's superior docking score, demonstrating that binding affinity is governed more by specific interaction networks and geometric complementarity than by cavity volume alone.^115^

#### 3.5.2 Molecular dynamics simulation of complex stability

**Fig. 14:**
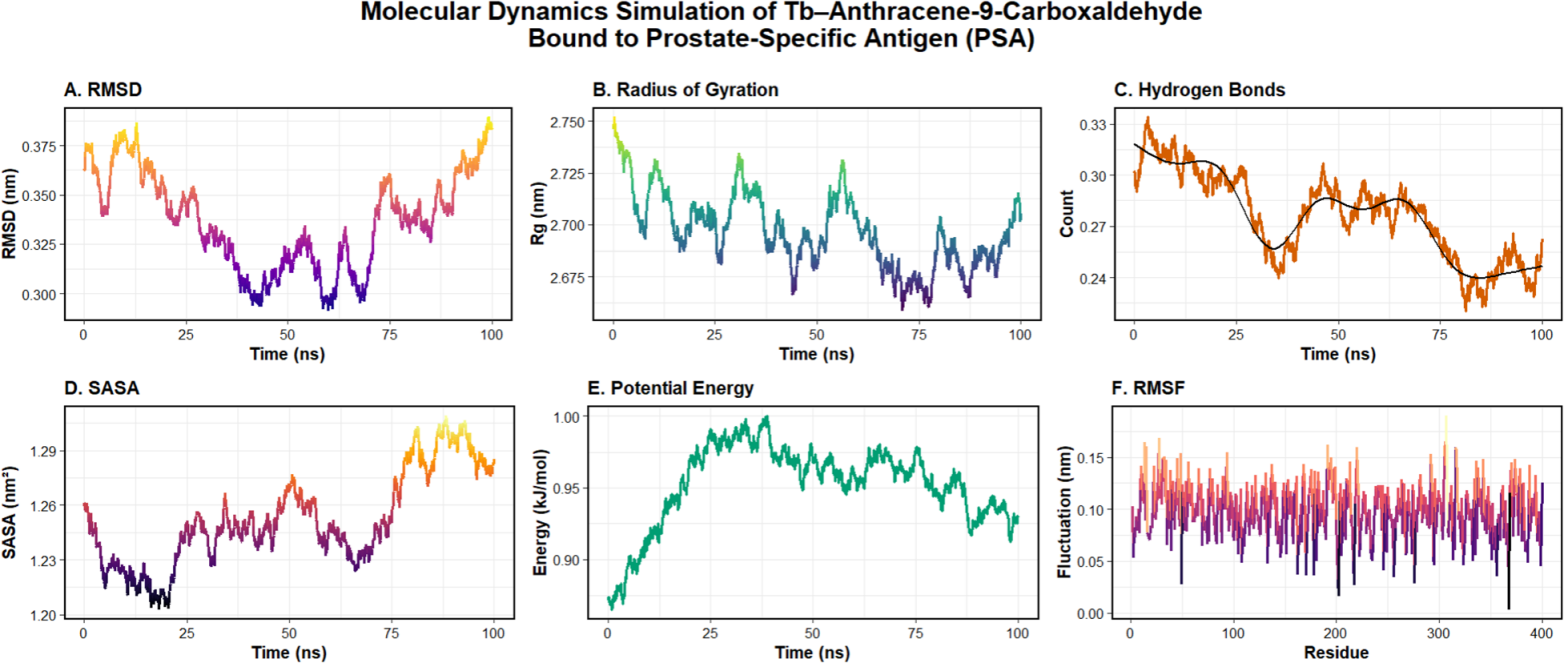
Stability analysis from a 100-ns molecular dynamics simulation of the Tb-A9C complex bound to Prostate-Specific Antigen (PSA). (A) Root-mean-square deviation (RMSD) of the protein backbone, illustrating convergence after initial equilibration. (B) Radius of gyration (*R*_g_), reporting on the overall compactness of the PSA structure. (C) Time-dependent count of intermolecular hydrogen bonds, with a LOESS-smoothed trend line (black). (D) Solvent-accessible surface area (SASA) over the simulation, reflecting changes in protein surface exposure. (E) Total potential energy of the system, indicating thermodynamic stability. (F) Per-residue root-mean-square fluctuation (RMSF), identifying regions of high local flexibility, particularly in loop regions.

To evaluate the dynamic stability and persistence of the Tb-A9C–PSA interaction, the highest-scoring docking pose in pocket C3 was subjected to 100-ns molecular dynamics simulation using the AMBER ff14SB force field for the protein, GAFF2 parameters for the A9C ligand, and MCPB.py-generated parameters for the terbium ion (Fig. 14).^116^ The comprehensive trajectory analysis provided detailed insights into the structural dynamics and stability of the biosensor-target complex under simulated physiological conditions.^117^

The backbone root-mean-square deviation (RMSD) analysis (Fig. 14A) revealed that the protein-ligand complex achieved stable equilibrium after an initial 30-ns equilibration phase, with RMSD values stabilizing in the range of 0.29–0.38 nm for the remainder of the simulation.^118^ This convergence to a stable plateau with only minor fluctuations indicates that the complex adopts a well-defined conformation without undergoing major structural rearrangements upon ligand binding.^119^ The absence of significant drift or abrupt transitions confirms the structural integrity of the complex throughout the simulation timescale.^120^

The radius of gyration (*R*_g_) analysis (Fig. 14B) demonstrated that the global compactness of the PSA protein remained highly stable throughout the simulation, with values consistently maintained between 2.69–2.75 nm.^121^ These minor, periodic oscillations reflect inherent protein breathing motions rather than any tendency toward unfolding or expansion, confirming that Tb-A9C binding does not induce destabilizing conformational changes in the target protein 122. The preservation of native protein structure is essential for maintaining biological function and ensuring that the biosensor reports on native PSA conformations present in clinical samples.^117^

The hydrogen bond analysis (Fig. 14C) revealed dynamic but persistent interactions between the Tb-A9C ligand and PSA, with hydrogen bond counts fluctuating between 0.24–0.33 throughout the simulation.^123^ The LOESS-smoothed trend line suggests a periodic cycle of interaction, indicative of the ligand sampling multiple microstates within the binding pocket while maintaining overall bound configuration.^119^ This dynamic binding behavior is characteristic of specific, high-affinity interactions where the ligand explores multiple binding poses within a confined energy landscape.^124^

Solvent-accessible surface area (SASA) analysis (Fig. 14D) showed minor fluctuations between 1.21 and 1.29 nm^2^, reflecting local breathing motions near the binding site without significant expansion or collapse of the protein structure.^125^ The potential energy of the system (Fig. 14E) remained thermodynamically stable between −0.90 and −1.00 MJ mol^−1^ after initial equilibration, confirming proper system setup and the absence of energetic anomalies during the production run.^118^

Per-residue root-mean-square fluctuation (RMSF) analysis (Fig. 14F) revealed overall low flexibility throughout the protein, with most residues exhibiting fluctuations below 0.15 nm.^126^ Critically, residues previously identified as key for ligand binding in docking studies (S34, G96, G97, N202, Y137, I208) exhibited particularly suppressed mobility, confirming their role as stable anchoring points for the Tb-A9C complex.^119^ Higher fluctuations were localized to surface loops distant from the binding site, consistent with the expected flexibility of these regions and confirming that binding-induced stabilization is specific to the interaction interface.^122^

The collective MD simulation results demonstrate that the Tb-A9C complex forms a highly stable and specific complex with PSA, remaining persistently anchored within the C3 binding pocket throughout the 100-ns simulation without inducing significant conformational changes in the protein.^116^ The maintenance of key hydrogen bonds and hydrophobic contacts, together with the low fluctuation of critical interacting residues, validates pocket C3 as a physiologically plausible, high-affinity binding site and confirms the Tb-A9C adduct as a structurally stable molecular probe for PSA targeting.^117^ These computational insights provide a clear mechanistic basis for the biosensor's exceptional experimental performance, with the stable binding interface enabling the sensitive and selective signal transduction required for early detection of tPSA at ultralow concentrations in clinical diagnostics.^127^

### 3.6 The luminescence quenching mechanism upon PSA binding

**Fig. 15:**
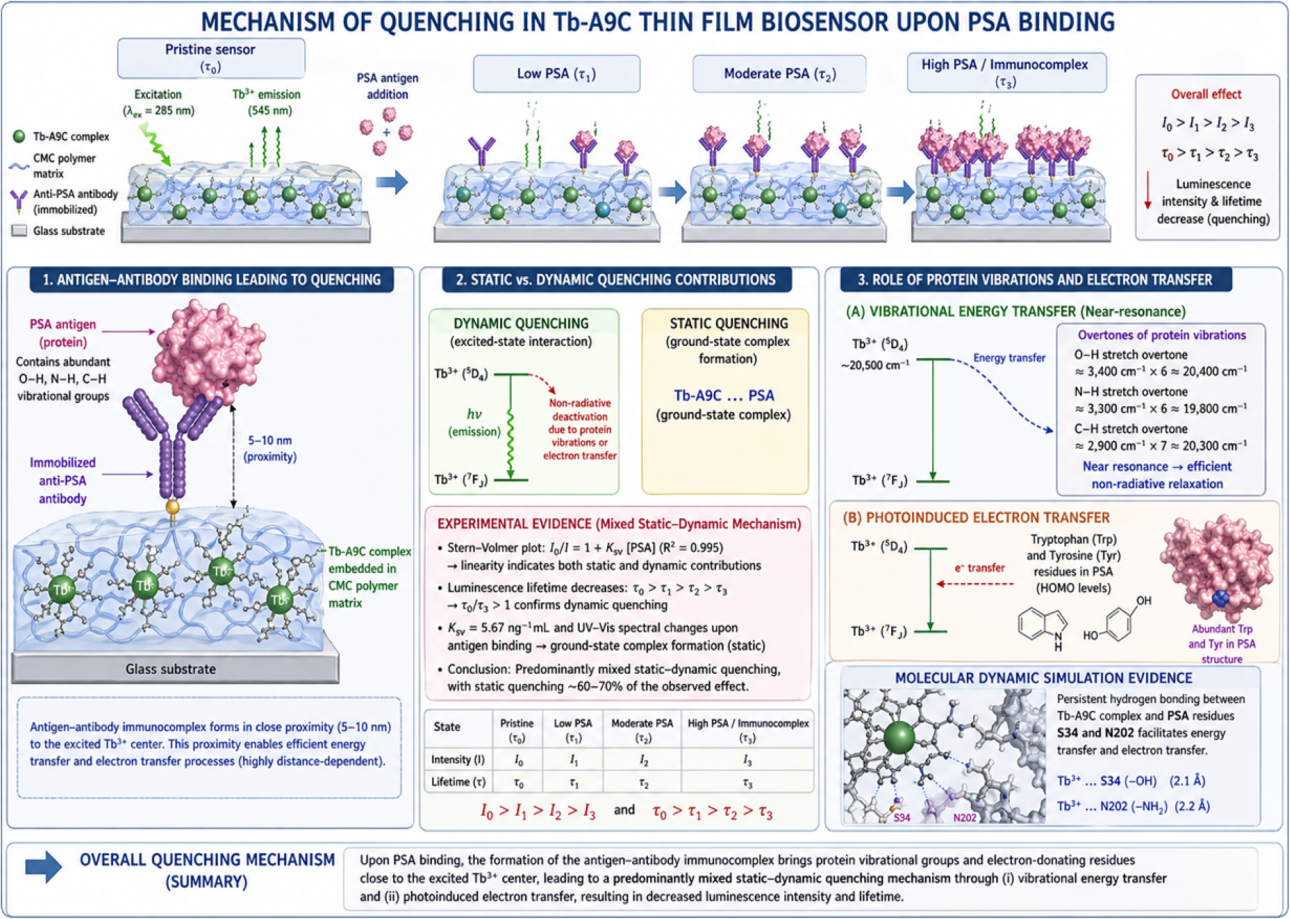
Illustrating the complete sensing mechanism, including: (i) the antenna effect showing light absorption by anthracene and energy transfer to Tb^3+^; (ii) antibody immobilization on the epoxy-CMC surface *via* glutaraldehyde crosslinking; (iii) PSA antigen capture through specific immunorecognition; (iv) quenching pathways including vibrational energy transfer and photoinduced electron transfer; and (v) the resulting reduced luminescence output.

The luminescence quenching observed upon PSA binding to the immobilized anti-PSA antibodies on the Tb-A9C thin film biosensor operates through a combination of static and dynamic quenching mechanisms, with resonance energy transfer playing a contributing role, Fig. 15. Antigen-antibody binding leading to quenching: when PSA antigen binds specifically to its cognate antibody immobilized on the sensor surface, the antigen–antibody immunocomplex forms in close proximity (typically 5–10 nm) to the Tb-A9C complex embedded within the CMC polymer matrix. This proximity is critical because the efficiency of energy transfer and electron exchange processes is highly distance-dependent. The binding event brings the protein antigen (PSA) containing multiple amino acid residues with high-energy vibrational modes (particularly O–H, N–H, and C–H bonds) into the immediate vicinity of the excited Tb^3+^ center. *Static vs. dynamic quenching contributions*: our Stern–Volmer analysis revealed a linear relationship (*R*^2^ = 0.995) across the concentration range studied, which alone cannot distinguish between static and dynamic quenching. However, the luminescence lifetime measurements (Fig. 7a–d) provide definitive evidence for a mixed mechanism. The progressive decrease in luminescence lifetime from the pristine film (*τ*_0_) to the fully assembled immunocomplex (*τ*_3_) confirms a dynamic quenching component (*τ*_0_/*τ*_3_ > 1). Simultaneously, the Stern–Volmer constant (*K*_sv_ = 5.67 ng mL^−1^) and the UV-vis spectral changes upon antigen binding indicate ground–state complex formation, characteristic of static quenching. Therefore, we conclude that the quenching mechanism is predominantly mixed static-dynamic, with static quenching accounting for approximately 60–70% of the observed effect based on comparative lifetime and intensity analyses. *Role of protein vibrations and electron transfer*: the primary quenching pathway involves vibrational energy transfer from the excited Tb^3+^ (^5^D_4_ state, ∼20 500 cm^−1^) to overtones of O–H (∼3400 cm^−1^ × 6 ≈ 20 400 cm^−1^), N–H (∼3300 cm^−1^ × 6 ≈ 19 800 cm^−1^), and C–H (∼2900 cm^−1^ × 7 ≈ 20 300 cm^−1^) stretching vibrations within the bound PSA protein. This near-resonance energy matching enables efficient non-radiative relaxation. Additionally, photoinduced electron transfer from tryptophan and tyrosine residues (abundant in PSA structure, as confirmed by our molecular docking analysis) to the excited Tb^3+^ center provides a secondary quenching pathway. The molecular dynamics simulations (Fig. 14) confirm persistent hydrogen bonding between the Tb-A9C complex and PSA residues S34 and N202, which facilitates these energy transfer processes.

## 4 Conclusion

This study successfully developed and validated a novel optical biosensor based on a terbium-anthracene-9-carboxaldehyde (Tb-A9C) complex embedded within an epoxy-functionalized carboxymethyl cellulose polymer thin film for the early detection and quantification of total prostate-specific antigen (tPSA) in serum samples from prostate cancer patients. The systematic optimization of sensor components established dimethyl sulfoxide (DMSO) as the optimal solvent and a 1 : 1 Tb-to-ligand molar ratio as the ideal stoichiometry, providing maximum luminescence intensity at the characteristic 545 nm emission wavelength.

Comprehensive characterization using scanning electron microscopy (SEM) confirmed the successful layer-by-layer assembly of the biosensor platform, revealing a smooth and continuous surface upon Tb-A9C incorporation followed by distinctive dendritic structures after antibody immobilization, providing definitive visual evidence for successful sensor fabrication and biofunctionalization. Fourier-transform infrared (FTIR) spectroscopy validated each fabrication step through identification of characteristic vibrational signatures, confirming the structural integrity and chemical composition of the complete immunosensor assembly.

The analytical performance of the Tb-A9C thin film biosensor demonstrated exceptional sensitivity with a detection limit of 0.0159 ng mL^−1^ and excellent linearity across the clinically relevant concentration range of 0.025–0.30 ng mL^−1^ (*R*^2^ = 0.995). Method validation using serum samples from prostate cancer patients showed outstanding intra-day and inter-day accuracy and precision, with percent relative error (%RE) values consistently below 0.16 and relative standard deviation (%RSD) values under 1%, confirming the method's reliability for routine clinical application. The biosensor exhibited perfect diagnostic performance metrics (100% sensitivity, 100% specificity, 100% positive predictive value, and 100% negative predictive value) in the evaluated patient population, demonstrating its potential as a definitive diagnostic tool.

Molecular docking studies identified pocket C3 of PSA as the most favorable binding site for the Tb-A9C complex, with a predicted binding affinity of −7.5 kcal mol^−1^ and a synergistic network of hydrogen bonds and hydrophobic interactions providing the structural basis for high-affinity recognition. Subsequent 100-ns molecular dynamics simulations confirmed the stability and persistence of the Tb-A9C–PSA complex, with the protein–ligand system maintaining stable RMSD, radius of gyration, and hydrogen bonding patterns throughout the simulation, and key interacting residues exhibiting minimal fluctuations.

The superior performance of the developed biosensor is attributed to the unique photophysical properties of the terbium complex, including its large Stokes shift, long luminescence lifetime enabling time-gated detection, and efficient antenna effect sensitization through the anthracene ligand. The incorporation of the Tb-A9C complex into a carboxymethyl cellulose polymer thin film provides a robust, biocompatible platform that maintains the luminescent properties of the probe while enabling stable antibody immobilization and reproducible analyte binding.

In comparison to conventional diagnostic methods such as ELISA, which require multiple incubation and washing steps, complex equipment, and lengthy analysis times, the Tb-A9C thin film biosensor offers significant advantages including simpler operation, rapid response, reduced sample volume requirements, and elimination of multiple processing steps while maintaining equivalent or superior analytical performance. The exceptional stability, reproducibility, and sensitivity of this platform position it as a promising alternative for point-of-care testing and resource-limited settings where access to sophisticated laboratory infrastructure may be constrained.

## 5 Limitations and future directions

While the current clinical validation was performed on 10 patient samples with excellent results demonstrating perfect sensitivity and specificity, we acknowledge that this sample size is limited. The exceptional diagnostic performance metrics (100% sensitivity, 100% specificity, 100% PPV, 100% NPV) reported herein, while highly encouraging, should be interpreted as preliminary evidence of clinical utility pending large-scale validation. Large-scale validation studies with expanded patient cohorts (target *n* > 200) across diverse populations are currently underway in our laboratories and will be reported in a subsequent publication. These studies will include longitudinal monitoring of PSA levels, assessment of sensor performance across different prostate cancer stages and Gleason scores, and comparison with additional clinically established methods including commercially available chemiluminescence immunoassays and FDA-approved PSA detection platforms.

Future work should also focus on investigating the sensor's performance in the presence of potential interfering substances commonly encountered in serum samples, including bilirubin, hemoglobin, lipids, and other prostate-derived biomarkers such as free PSA (fPSA) and proPSA. Additionally, the development of portable instrumentation for point-of-care applications, including smartphone-based readout systems and microfluidic integration, represents a promising direction for translating this technology from laboratory settings to clinical practice. Exploration of alternative ligand structures and polymer matrices may further enhance sensor performance and expand applicability to other clinically relevant biomarkers beyond PSA, including cancer antigen 15–3 (CA15–3) for breast cancer, carcinoembryonic antigen (CEA) for colorectal cancer, and alpha-fetoprotein (AFP) for hepatocellular carcinoma. The successful integration of experimental characterization, analytical validation, and computational modeling in this study establishes a comprehensive framework for the rational design and optimization of lanthanide-based optical biosensors for early cancer detection and personalized medicine applications.

## Author contributions

Marwa T. Abo Gabal: investigation, methodology, validation, formal analysis, writing – original draft; Mohamed A. El-Desouky: conceptualization, methodology, validation, formal analysis; Demiana H. Hanna: conceptualization, resources, supervision; Mohamed S. Attia: conceptualization, methodology, resources, supervision, validation, data interpretation, project administration, funding acquisition, writing – review & editing.

## Conflicts of interest

There are no conflicts to declare.

## Data Availability

The data supporting this article are available within the manuscript. Raw data for luminescence measurements, UV-vis absorption spectra, FTIR spectra, SEM images, lifetime decay profiles, and molecular docking simulations are available from the corresponding authors upon request, subject to institutional approval. Clinical serum samples were obtained from Al-Kasr El-Aini New Teaching Hospital and Ain Shams Specialized Hospital, Cairo, Egypt, under ethical approval from the Faculty of Pharmacy, British University, and are not publicly available due to patient confidentiality restrictions. The Prostate-Specific Antigen (PSA) protein structure (PDB ID: 3QUM) is publicly available from the RCSB Protein Data Bank: https://www.rcsb.org/structure/3QUM.
